# Pregnane X receptor (NR1I2) deficiency in mice reveals context-dependent regulation of inflammatory homeostasis

**DOI:** 10.3389/fimmu.2026.1761552

**Published:** 2026-05-12

**Authors:** Salah Edden Amini

**Affiliations:** Department of Molecular Medicine, Institute of Basic Medical Sciences, Faculty of Medicine, University of Oslo, Oslo, Norway

**Keywords:** gut microbiota, immunometabolism, inflammation, inflammatory bowel disease, mouse models, NR1I2, pregnane X receptor (PXR, xenobiotic receptor)

## Abstract

Nuclear receptor subfamily 1 group I member 2 (NR1I2; mouse ortholog Nr1i2), also known as the pregnane X receptor (PXR) or steroid and xenobiotic receptor (SXR), is a ligand-activated transcription factor classically known for its role in xenobiotic metabolism and detoxification. Beyond these canonical functions, accumulating evidence identifies NR1I2 as a key regulator of inflammation and intestinal homeostasis, particularly in inflammatory bowel disease (IBD), where dysregulated inflammatory responses are central to disease pathogenesis and NR1I2 expression is often reduced. In this review, we integrate data from whole-body and tissue-specific Nr1i2-deficient mouse models, as well as knockdown and ligand-based approaches, to define the role of Nr1i2 in intestinal inflammation under basal and pathological conditions. These studies collectively show that whole-body Nr1i2 deficiency disrupts intestinal homeostasis, impairs barrier integrity, and enhances innate immune activation, whereas tissue-restricted deletion, especially within the epithelium, frequently fails to reproduce these phenotypes, underscoring the importance of coordinated Nr1i2 activity across multiple cellular compartments. Across experimental models, most often Nr1i2 deficiency commonly exacerbates intestinal injury, particularly in response to microbial toxins or chemical damage, however attenuated inflammatory responses have been reported in selected metabolic or injury contexts. Conversely, pharmacological activation of Nr1i2 confers protective and restorative effects in a ligand-, tissue-, and context-dependent manner. Together, these findings establish NR1I2 not as a simple anti-inflammatory switch, but as an immune–metabolic integrator that coordinates xenobiotic detoxification, microbial-derived signal sensing, and restraint of innate inflammatory pathways. This review provides a conceptual framework for future studies aimed at elucidating the cell- and context-specific functions of NR1I2 and for guiding the development of targeted therapeutic strategies for intestinal inflammatory disorders.

## 1 Introduction

### 1.1 Nuclear receptor 1I2 (NR1I2/PXR)

Nuclear receptor subfamily 1 group I member 2 (NR1I2; mouse ortholog Nr1i2), also known as the pregnane X receptor (PXR) or steroid and xenobiotic receptor (SXR), is a ligand-activated transcription factor belonging to the nuclear receptor (NR) superfamily (1–3). The *NR1I2* gene is composed of nine exons. It is located on chromosome 3 (3q13.33) in humans, spanning 38kb, and in mice, *Nr1i2* is located on chromosome 16 (16 B3; 26.64), spanning 46.5kb (4, 5). *NR1I2/Nr1i2* mRNA is highly expressed in the liver, small intestine, colon, rectum, and bladder (2, 5, 6). However, its expression in the lungs, stomach, kidney, peripheral blood monocytes, uterus, ovaries, adrenal glands, bone marrow, and some brain regions is moderate or low (5, 7).

The NR1I2 protein is a 50-kDa molecule that shares some structural features with other NR. It has an N-terminal conserved DNA-binding domain (DBD), activation function domain 1 (AF-1), hinge region, and the C-terminal ligand-binding domain (LBD) with an activation function 2 (AF-2) (8, 9). The hinge region connects the DBD and the LBD. NR1I2 interacts with the promoter region of target genes via the two zinc fingers on the DBD (2, 8). NR1I2 orthologues across different species share more than 94% amino acid identity in the DBD, but homology in the LBD is significantly lower (10). The NR1I2-LBD has a short helix (αAF) at its end, essential for the AF-2 region to recruit transcriptional co-regulators (6).

The NR1I2 has a large, flexible, and dynamic LBD that can accommodate various hydrophobic ligands with different structural and physicochemical properties (3, 11). These ligands may include natural steroids such as progesterone and corticosterone, bile acids (BA) like lithocholic acid (LCA), synthetic drugs such as pregnenolone 16α-carbonitrile (PCN) and dexamethasone, antibiotics like rifampicin, microbial metabolites, herbal compounds, and environmental agents (2, 12, 13). Co-activator and co-repressor proteins are important components in transcriptional gene regulation machinery. Specific co-activator proteins that collaborate with NR1I2 to modulate target-gene expression include steroid receptor coactivators (SRC1, SRC2, p300), and peroxisome proliferator-activated receptor gamma coactivator 1 alpha (PGC1α) (1, 14, 15). The co-repressor proteins known to associate with NR1I2 physically include the nuclear receptor co-repressor (NCoR) and silencing mediator for retinoid and thyroid receptors (SMRT), histone deacetylase 3 (HDAC3), and heat shock protein 90 (HSP90) (2, 15, 16). The activity of NR1I2 is also regulated via crosstalk with other nuclear receptors (NR1I3, VDR, HNF4A, NR1H3, NR1H4, PPAR) or transcription factors (CREB, FOXO, NFKB) by sharing or competing for the binding of a co-regulatory molecule or response element on the target gene (12, 17–19). In addition, several post-translational modifications of NR1I2 have been observed or inferred that modulate NR1I2 biological activity and sub-cellular location, including phosphorylation (20–23), ubiquitylation, SUMOylation, acetylation, and poly(ATP-ribosyl)ation (23–25). When the ligand enters the cell, it binds to the LBD at the C terminus of NR1I2, causing a conformational change that disengages the co-repressor. The activated NR1I2 translocates from the cytoplasm to the nucleus. It forms a heterodimer with retinoid X receptor (RXR), which recruits the co-activator in the nucleus and aggregates on the NR1I2-responsive element. This aggregation loosens the nuclear chromatin structure and activates transcription, thereby inducing the expression of target genes.

NR1I2 is considered a master xenobiotic sensor that predominantly regulates the expression of genes related to phase I and II metabolic enzymes and drug transporters critical for xenobiotic and endobiotic elimination. Upon activation, it regulates downstream genes, like enzymes of the mitochondrial cytochrome P450 family (*CYP3A* and *CYP2B*), transferases (*GST1*), and ABC transporters (*MDR*) that are necessary for detoxification (26, 27). NR1I2 also plays a crucial role in regulating energy metabolism, cell cycle, apoptosis, and various disorders like cholestasis, steatosis, immune disorder, and inflammatory bowel disease (5, 12, 28–32). Beyond xenobiotic detoxification, NR1I2 has emerged as a key interface between epithelial metabolism, microbial-derived ligands, and innate immune signaling, making *Nr1i2*-deficient mouse models particularly informative for dissecting its role in intestinal inflammation and IBD (33–38).

### 1.2 Inflammation

Inflammation is an adaptive defense mechanism that helps fight against infections and endogenous signals such as tissue injury (39–41). It is a non-specific and immediate response to remove injurious stimuli and initiate healing. Inflammation is crucial for maintaining homeostasis (42, 43). There are three types of inflammation: acute, chronic, and subacute. Acute inflammation occurs immediately after an injury or infection. It involves soluble mediators such as cytokines, acute phase proteins, and chemokines that cause neutrophils and macrophages to migrate to the inflamed area (42, 44, 45). A successful acute inflammatory response results in the elimination of infectious agents and necrotic cells, followed by a resolution and repair phase mediated by tissue-resident and recruited cells, and a switch from pro-inflammatory to anti-inflammatory mediators (42). However, when acute inflammation fails to settle, it can develop into subacute and then chronic inflammation, which is characterized by the migration of T lymphocytes and plasma cells to the site of inflammation. The persistence of chronic inflammation causes tissue damage, fibrosis, cancer, and other chronic inflammatory diseases (39–41, 43, 44, 46).

Inflammation occurs when cells detect structures found on pathogens (pathogen-associated molecular patterns) or endogenous stress signals (danger-associated molecular patterns) using pattern-recognition receptors (PRRs) like toll-like receptors (TLRs) (39, 44, 47, 48). PRRs are mostly found in myeloid cells such as monocytes, macrophages, neutrophils, and dendritic cells, but can also be found in lymphocytes, fibroblasts, and epithelial cells. The transmission of PAMPs and DAMPs is then mediated by myeloid differentiation 88 (MyD88) and TLRs (47, 48). This leads to a specific cascade of signaling that causes transcription factors like NF-κB to move into the nucleus. This activation triggers an inflammatory response which can cause the release of pro-inflammatory cytokines like tumor necrosis factor-α (TNF-α), and interleukins (IL-1, IL-6, IL-8), colony-stimulating factor (CSF), as well as enzymes like cyclooxygenase-2 (COX-2) and proteins like matrix metalloproteinases (MMP) (39, 43, 49). Cells have also mechanisms that help to shut down the inflammatory response and return to homeostasis. This active process involves the functional reprogramming of cells by producing mediators that can inhibit inflammation. The cytokines IL-10 and TGF-β suppress the production of pro-inflammatory cytokines, while cleaved extracellular domains of cytokine receptors, such as soluble TNFR and IL-1R, limit inflammation by binding and neutralizing their respective cytokines. In addition, receptor antagonists like IL-1Ra, bind IL-1R without inducing an intracellular signal, thus inhibiting the biological activity of the interleukins IL-1α and IL-1β (43, 49).

Dysregulated inflammatory responses play a pivotal role in a myriad of human disorders, spanning infectious and autoimmune conditions to neurological, cardiovascular, renal, and hepatic ailments (46). This dysregulation can manifest as chronic inflammatory diseases, such as inflammatory bowel disease (IBD), notably ulcerative colitis (UC) and Crohn’s disease (CD) (50). These conditions are characterized by sustained mucosal inflammation driven by adaptive and innate immune responses (51–53). Furthermore, the pathophysiological presentation of IBD often extends beyond the gastrointestinal tract, giving rise to extra-intestinal manifestations. These complications stem from the persistent systemic inflammatory milieu induced by IBD, which disrupts various signaling pathways and modulates the expression of regulatory molecules such as cytokines and mRNAs (46, 50, 54). The cascading effect of inflammatory cytokines triggers a cycle of intestinal and extra-intestinal tissue damage and perpetuates inflammation, thereby contributing to the progression of the disease (55–57).

It is widely recognized that the expression of xenobiotic nuclear receptors as the *NR1I2* is downregulated during inflammatory processes (2, 34, 35, 58, 59; L. 31; W. 33). Specifically, in colon colitis, *NR1I2* expression shows a reduction, while in patients with Crohn’s disease, it remains unaffected (60) or decreases (61). Notably, studies indicate a strong inverse correlation between epithelial *NR1I2* expression in the colon and levels of *NFκB* and *IL8* in colon biopsies from individuals with Crohn’s disease (62). Experimental findings demonstrate that the addition of TNFα to CaCo-2 cell monolayers leads to diminished *NR1I2* expression and upregulation of *IL8*, suggesting that TNFα-mediated inhibition of *NR1I2* expression occurs via the NFKB pathway (61). Additionally, in mice models of ulcerative colitis reduced Nr1i2 protein expression levels are observed in the liver (63, 64). *Nr1i2* expression is also downregulated in the intestine of mice treated with LPS (Y. H. 65) and in transgenic mice with constitutive expression of Tnf (61).

To further elucidate the role of Nr1i2 in inflammatory processes, numerous studies have employed various *Nr1i2-deficient mouse* models. These models provide valuable insights into the impact of *Nr1i2* deficiency on inflammatory responses and associated pathologies. By utilizing *Nr1i2-deficient mouse* models, researchers can explore the downstream consequences of Nr1i2 downregulation in various inflammatory contexts, shedding light on the intricate mechanisms underlying Nr1i2-mediated effects *in vivo*.

## 2 Nr1i2-deficient mouse models

### 2.1 Whole-body knockout

The characteristics of whole-body Nr1i2 knockout models are summarized in **Table 1**.

**Table 1:** Summary of whole-body knockout models.

Whole-body knockout model	Targeted exon(s)	Deletion validated in	Cyp3a expression
*Xie* et al. model	2^nd^ + 3^rd^	Liver Small intestine	Not altered
*Staudinger* et al. model	1^st^	Liver Small intestine	Increased
*Luan* et al. model	3^rd^	Kidney	Not reported

#### 2.1.1 *Xie* et al. model

Mouse *Nr1i2* genomic DNA was extracted by screening a 129/Sv library with a *Nr1i2* complementary DNA probe. Subsequently, a targeting vector was engineered by substituting the second and third exons of *Nr1i2* with the phosphoglycerate kinase I – Neomycin (PGK-Neo) selection marker. This introduced cassette harbors the neomycin phosphotransferase gene controlled by the phosphoglycerate kinase I (PGK) promoter, alongside a negative selection marker (PGK-TK), specifically the herpes simplex virus TK gene (66). Following transfection, single J1 embryonic stem (ES) cell clones that exhibited resistance to G418 and ganciclovir were meticulously screened for the desired homologous recombination through Southern blotting. *Nr1i2*^+/-^ ES cells were then microinjected into C57BL6/J blastocysts, which were subsequently transplanted into the uteri of pseudopregnant ICR mice. Chimeric male progeny resulting from this procedure were subsequently bred with C57BL6/J females. The germline transmission of the disrupted allele was identified in agouti progeny through both Southern blot and polymerase chain reaction (PCR) analyses (66). The resultant mutant allele harbors a deletion encompassing two exons, including amino-acid residues 63–170 within the DNA-binding domain. The absence of *Nr1i2* expression in the two primary *Nr1i2*-expressing tissues, liver, and small intestine, confirmed the deficiency of *Nr1i2* alleles. The *Nr1i2^−/−^* mice exhibit both viability and fertility. Despite this, the basal expression of Cyp3a remains unaltered in the absence of *Nr1i2*. However, the induction of *Cyp3a* genes in response to prototypic rodent-specific *Cyp3a* inducers, pregnenolone-16α-carbonitrile (PCN), and dexamethasone (DEX), is no longer observed (66).

#### 2.1.2 *Staudinger* et al. model

Three genomic clones harboring the mouse *Nr1i2* locus were identified through screening a 129/Sv genomic library using a cDNA probe derived from mouse *Nr1i2* cDNA. Subsequently, a targeting vector was constructed by replacing a segment from the first exon of *Nr1i2* with a PGK-Neo selection marker, alongside a negative selection marker (PGK-TK) (67). Mouse R1 ES cells originating from the 129-Sv strain were cultivated and subjected to electroporation. ES cells with plasmid integration via homologous recombination with the native *Nr1i2* locus were discerned through Southern blot analysis of DNA extracted from individual ES cell colonies. *Nr1i2*^+/-^ ES cells containing the accurately targeted locus were introduced into C57BL/6J blastocysts, leading to the generation of chimeric males that were subsequently bred with C57BL/6 females. Progeny were screened via Southern blot analysis of DNA extracted from tail biopsies to identify individuals heterozygous for the mutant *Nr1i2* allele. These heterozygous offspring were then interbred to produce mice homozygous for the *Nr1i2* mutation and wild-type littermate controls (67). Confirmation of *Nr1i2* allele disruption was achieved by the absence of *Nr1i2* expression in the primary *Nr1i2*-expressing tissues, namely the liver and small intestine. *Nr1i2^−/−^* mice exhibited viability and fertility. Notably, basal levels of *Cyp3a11* mRNA were elevated in *Nr1i2^−/−^* mice, revealing an unexpected role for *Nr1i2* in repressing basal *Cyp3a11* expression. This effect of *Nr1i2* deletion on basal *Cyp3a11* expression was not observed in the *Xie* model, although the underlying cause for this discrepancy remains unknown. Nevertheless, the induction of the *Cyp3a11* gene in response to PCN and DEX was abolished (67).

#### 2.1.3 *Luan* et al. model

This model was developed through a partnership with Shanghai Model Organisms. Initially, genomic DNA from Mouse *Nr1i2* was isolated by screening a 129/Sv library using a complementary DNA probe specific to *Nr1i2*. Subsequently, a targeting vector was created, replacing the third exon of *Nr1i2* with a PGK-Neo selection marker, followed by a negative selection marker (PGK-TK) (68). Mouse ES cell clones were then screened via Southern blotting to confirm designated homologous recombination. *Nr1i2^+/-^* ES cells were introduced into C57BL6/J blastocysts, leading to chimeric male progeny, which were later crossed with C57BL6/J females. Germline transmission of the disrupted allele was confirmed in agouti progeny through Southern blot and polymerase chain reaction (PCR) analysis (68). The absence of *Nr1i2* expression in the kidney confirmed the depletion of *Nr1i2* alleles. Remarkably, *Nr1i2^−/−^* mice exhibited both viability and fertility. However, this model did not demonstrate Cyp3a expression (68).

### 2.2 RNA interference knockdown

The characteristics of Nr1i2 knockdown models are summarized in **Table 2**.

**Table 2:** Summary of RNA interference knockdown models.

RNA interference model	Mechanism	Knockdown assessed in	Cyp3a expression
shRNA knockdown	Liposome-shRNA	Liver	Not altered
siRNA knockdown	3 x siRNA Mix	Liver Colon	Decreased

#### 2.2.1 shRNA knockdown model

The *Nr1i2* knockdown (*Nr1i2-^KD^*) mice were generated through transfection of *Nr1i2*-targeting short hairpin RNAs (*Nr1i2* shRNA) on PCI(neomycin) plasmid (69). These shRNAs designed to reduce *Nr1i2* expression were driven by a mouse H1 promoter (cloned from HepG2 cells, supplement A). The preparation of the cationic liposome-shRNA complex involved combining the shRNA with Lipofectamine™ 2000 (69). Subsequently, this liposome-shRNA mixture was injected via the C57BL/6 mouse tail vein. In contrast, the negative control (*Nr1i2-^WT^*) mice received only the blank plasmid PCI mixed with Lipofectamine™ 2000 (69).

#### 2.2.2 siRNA knockdown model

Three target-specific short interfering RNAs each composed of 20–25 nucleotides were employed to suppress *Nr1i2* gene expression (*Nr1i2* siRNA) (70, 71). These synthetic *Nr1i2* siRNAs, featuring 2′-O-Methyl oligo (OMe) modification, were combined and administered *in vivo* using a modified hydrodynamic transfection method. In this approach, siRNA dissolved in PBS was rapidly injected into the tail vein of BALB/c mice (*Nr1i2-^KD^*). The *Nr1i2^-WT^* mice received an equivalent volume of non-targeting siRNA (siRNA-NC) as a control. These siRNA injections were repeated once every 3 days (70, 71).

### 2.3 Tissue-specific knockout

To generate tissue-specific knockout, mice bearing *Nr1i2* flox alleles (*Nr1i2^fl/fl^*) were employed. This involved utilizing mouse embryonic stem cell clones containing the conditional *Nr1i2* flox allele obtained from the International Knockout Mouse Consortium (EUMMCR, Nr1i2tm1a(EUCOMM)Wtsi, EPD0141_1_G04) to generate *Nr1i2^fl/fl^* mice on C57BL/6 background. The characteristics of tissue-specific Nr1i2 knockout models are summarized in **Table 3**.

**Table 3:** Summary of tissue-specific knockout models.

Tissue-specific knockout model	Cre driver line	Deletion validated in	Cyp3a expression
Hepatocyte (*Nr1i2*^ΔHep^)	Albumin-Cre	Liver	Not reported
Intestinal epithelium (*Nr1i2^ΔIEC^*)	Villin-Cre	Small intestine Colon	Not reported
Intestinal fibroblast (*Nr1i2^ΔCol1a2^*)	Col1a2-CreERT2*	Colonic fibroblasts	Not reported

(*) indicates that Cre-mediated recombination was induced by intraperitoneal 4-OHT injections.

#### 2.3.1 Hepatocyte-specific *Nr1i2* knockout

*Nr1i2^fl/fl^* mice were subsequently crossed with Albumin-Cre transgenic mice to generate hepatocyte-specific *Nr1i2*-deficient mice (*Nr1i2^ΔHep^*) (72). Notably, the *Nr1i2^fl/fl^* and *Nr1i2*^ΔHep^ mice used in studies were littermates with *Nr1i2*^ΔHep^ mice carrying heterozygous knock-in for Albumin-Cre (72–74). PCR analysis of genomic DNA extracted from major tissues confirmed the specificity of Albumin-Cre-mediated to the liver (72).

#### 2.3.2 Intestinal epithelial-specific *Nr1i2* knockout

*Nr1i2^fl/fl^* mice were bred with Villin-Cre mice (75) to generate intestinal epithelial-specific *Nr1i2* knockout (*Nr1i2^ΔIEC^*) (73). Throughout experiments involving *Nr1i2^ΔIEC^* mice also included their corresponding *Nr1i2^fl/fl^* littermates as controls (38, 73, 74, 76). The specific depletion of *Nr1i2* alleles in the intestines was confirmed by the absence of *Nr1i2* in the duodenum, jejunum, ileum, and colon of *Nr1i2^ΔIEC^* mice, while *Nr1i2* expression in other major tissues remained similar between *Nr1i2^ΔIEC^* and *Nr1i2^fl/fl^* mice (38, 73, 74, 76).

#### 2.3.3 Intestinal fibroblast-specific *Nr1i2* knockout

Col1a2-CreERT2 (Tg(Col1a2-cre/ERT,-ALPP)7Cpd/J) mice, bred on the C57BL/6 background, were obtained from The Jackson Laboratory (38). Crossing *Nr1i2^fl/fl^* mice with Col1a2-CreERT2 mice resulted in the generation of intestinal fibroblast-specific *Nr1i2* knockout Col1a2-CreERT2-Nr1i2 (*Nr1i2^ΔCol1a2^*) mice (38). To induce Cre activity in male *Nr1i2^ΔCol1a2^* mice, intraperitoneal injections of 1 mg of 4-hydroxytamoxifen (4-OHT) were administered several times (initially, 3 injections to induce, followed by 1 injection weekly to maintain). The knockdown of *Nr1i2* following 4-OHT treatment was confirmed by assessing Nr1i2 protein expression in freshly isolated colon fibroblasts (38). Control experiments involving *Nr1i2^ΔCol1a2^* mice included their corresponding *Nr1i2^fl/fl^* littermates (38).

## 3 Study of the *Nr1i2* role in inflammation

### 3.1 Basal condition

Nr1i2 has been shown to help maintain basal intestinal homeostasis, and barrier integrity and to regulate gut inflammation. Whole-body *Nr1i2* knockout (*Nr1i2^−/−^*) mice had a higher intestinal weight-to-length ratio, a marker of tissue edema (77, 78), and intestinal barrier permeability (36). Histological examination of the mucosa of *Nr1i2^−/−^* small intestines showed significant increases in inflammation markers as indicated by a diminution of villus-crypt ratio, marked neutrophil infiltration, and increased myeloperoxidase (MPO) enzyme activity (36, 69, 79). Moreover, transmission electron microscopy analysis of *Nr1i2^−/−^* intestinal epithelial cells confirmed this inflammatory profile by showing loosely packed shorter microvilli (36). Further, it revealed a reduction in digestive enzymes (sucrose, lactase, maltase, dipeptidyl peptidase) and alkaline phosphatase (ALP) activities. These latter findings support a disturbance in the digestion and nutrient absorption capacity of *Nr1i2^−/−^* mice (36). However, classic inflammatory bowel disease (IBD) markers such as crypt abscesses, granulomata, or villous blunting, were not observed, and no significant changes in proliferation and apoptosis of the epithelium were identified in the intestine of *Nr1i2^−/−^* mice (36, 79). Furthermore, intestinal barrier integrity molecular marker analysis showed that the tight-junction, adherens-junction, and gap-junction intercellular complex in *Nr1i2^−/−^* mice intestinal epithelium was more diffuse with densely interconnected stranding and transcripts (Tjp1, Cldn, Ocln, Cdh1 and Gja1) expression were markedly diminished, reflecting defects in the intestinal barrier (28, 36). Additionally, the *Nr1i2^−/−^* mice showed significant downregulation of mRNA markers involved in anti-inflammatory (Il10 and Tgfb1) and antimicrobial (Muc2 and Lyz1) function, together with a concomitant increase in pro-inflammatory cytokine mRNA, especially NF-κB target genes like *Tnf*, *Il1b, Il6 and Ptgs2* (36, 79–81). Finally, toll-like receptors (Tlr4 and Tlr2), known as endotoxin receptors, and downstream pathway kinase (Mapk1 and Mapk8) activation was enhanced in *Nr1i2^−/−^* mice (36).

In addition to the role of Nr1i2 in regulating intestinal integrity and inflammation, Nr1i2 appears to modulate intestinal microbiota, which is age (82) and sex (Y. S. 82, 83) dependent. In young and adult *Nr1i2^−/−^* mice of both sexes, the lack of Nr1i2 increased the pro-inflammatory bacteria (*Helicobacteraceae, Helicobacter…*) richness, BA-deconjugating *Lactobacilli* that generate toxic unconjugated BAs. It decreased the anti-inflammatory microbiota (*A.muciniphila, Muribaculaceae…*), which together are linked to inflammation, oxidative stress, and cytotoxicity (84). Interestingly, these findings were found in male and female *Nr1i2^−/−^* mice (84). However, these results were not demonstrated in Zhou’s study where no difference in the pro-inflammatory *Helicobacter* colony was found (79). This difference could be explained either by the difference in the whole-body *Nr1i2* knockout mouse models (Staudinger or Xie model) respectively used, or either by the samples (feces or intestine) respectively analyzed, or housing conditions.

All these studies using different *Nr1i2^−/−^* mouse models revealed the adverse effects of *Nr1i2* depletion in basal intestinal homeostasis, barrier integrity, and gut inflammation. However, neither the histological observances (38, 79), nor the difference in inflammation marker (Tnf, Il1b and Il6) (79, 81) was demonstrated in the colon of *Nr1i2^−/−^*, nor even in the small intestine (Tlr4 and Il6) of the prenatal or early postnatal *Nr1i2^−/−^* mice (28). Nevertheless, similar histological damages were observed in extra-gut tissues like in the liver of shRNA whole-body silenced *Nr1i2* (*Nr1i2^-KD^*) mice (69), but not in the liver (79), spleen and lymph nodes (85) of *Nr1i2^−/−^* mice. In addition, the pro-inflammatory genes were elevated in the liver (*Il1a, Il1b, Il2, Il6 and Il15*) (80; C^79^), in primary hepatocytes (*Il1b, Il6, Tnf, Il1rn and Socs3*) (79, 86) and T lymphocyte (*Ifng*) (85) of *Nr1i2^−/−^* mice, but not in neurons (*Nlrp3*) (87).

Finally, our latest study is the only one that investigated the effect of tissue-specific *Nr1i2* knockout by exploring intestinal epithelial-specific *Nr1i2* knockout (*Nr1i2^ΔIEC^*) in intestinal homeostasis and inflammation in basal conditions (76). Compared to their control mice (*Nr1i2^fl/fl^*), *Nr1i2^ΔIEC^* shows the normal length of the small intestine, colon, and extra-intestinal tissues’ weight. The intestinal permeability and histological analysis (villus length, crypt depth, number of goblets, and Paneth cells) of *Nr1i2^ΔIEC^* mice were unaffected (76). Moreover, *Nr1i2^ΔIEC^* did not show downregulation of tight- and adherens-junction (*Tjp1*, *Cldn3*, and *Ocln*) and nor upregulation pro-inflammatory markers (*Tlr4*, *Myd88*, *Nlrp3*, *Casp1*, *Il1b*, *Il6*, *Il18* and *Ptgs2*) except *Il6* in the duodenum where it was upregulated (76). Tissue-specific *Nr1i2* knockout (*Nr1i2^Δ^)* does not globally affect the whole inflammation and intestinal integrity.

In conclusion, whole-body *Nr1i2* knockout (*Nr1i2^−/−^*) mice and not tissue-specific *Nr1i2* knockout (*Nr1i2^Δ^*) mice showed intestinal homeostasis disturbance characterized by histological damages, barrier integrity disorder, higher basal inflammation markers, and microbiota dysbiosis, especially in adult small intestine. Taken together, basal whole-body Nr1i2 expression appears important for intestinal homeostasis; however, the relative contribution of epithelial versus non-epithelial compartments and the precise molecular sequence linking detoxification programs to innate immune restraint remain incompletely resolved.

### 3.2 *Nr1i2* ligands treatment in basal condition

Since the *Nr1i2* deficiency generates basal inflammation while the basal expression of Nr1i2 plays an anti-inflammatory role, its activation would promote this protective effect. *In vivo* treatment with the Nr1i2 mouse ligand pregnenolone 16α-carbonitrile (PCN) further inhibited the expression of NF-κB target genes (*Tnf, Il1b, Il6*, and *Ptgs2*) (79). It decreased the presence of pro-inflammatory bacteria (*Dorea sp, Blautia sp*) (84) in control mice (*Nr1i2^fl/fl^*) intestine, but had no significant effect in *Nr1i2^−/−^* mice. A similar anti-inflammatory result was demonstrated in the expression of NF-κB target genes in the liver of control mice (*Nr1i2^fl/fl^*) (C. 79) treated with PCN. However, mice treated with HIV drug and Nr1i2 potent agonist, efavirenz, had liver enlargement and increased hepatic steatosis, increased plasma alanine aminotransferase (ALT) and aspartate transaminase (AST) levels, and elevated hepatic inflammation gene (*Ccl2*) in a Nr1i2*-*dependent manner. Deficiency of Nr1i2 abolished the impact of efavirenz in *Nr1i2^ΔHep^* and *Nr1i2^−/−^* mice (72). In addition, primary cultured hepatocytes (PCHs) treatment with PCN produced small but statistically significant increases in the levels of pro-inflammatory genes (*Tnf, Il1b, Il1rn*, and *Il6*) in PCH from control mice, and this effect was absent in the treated *Nr1i2^−/−^* PCHs (86). Finally, in T lymphocytes, PCN activation decreased the level of phosphorylated NF-κB p65, inhibited *Il2ra* expression and *Ifng* synthesis, and increased *Socs1* expression in an Nr1i2-dependent manner (85).

To conclude, few studies have extensively addressed the effect of Nr1i2 activation using whole-body and/or tissue-specific *Nr1i2* knockout. Nr1i2 activation shows controversial results depending on the agonist and the *in vivo* or *in vitro* models.

### 3.3 Under the inflammatory conditions

Previously we summed up that many studies demonstrated that Nr1i2 expression plays an important role in maintaining basal intestinal homeostasis and constitutes an anti-inflammatory barrier. In the following, we explore what is established about the role of Nr1i2 under induced inflammatory conditions.

#### 3.3.1 Bacterial toxins induction

*Clostridium difficile* toxin-induced injury (*Tcd A/B*) experiments were performed to assess the role of the Nr1i2 in inflammation. While *Nr1i2^+/+^* mice did not show substantial morbidity after 4 hours, *Nr1i2^−/−^* mice were extremely susceptible to toxin exposure, reaching defined endpoints after 2 hours, and failing to reach the predetermined 4-hour experimental endpoint. A similar dramatic survival result was shown in the endotoxin shock model, whereby systemic Lipopolysaccharides (LPS) induce a sepsis-like syndrome. *Nr1i2^−/−^* mice treated with LPS had significantly worse survival than their *Nr1i2^+/+^* counterpart (36). However, epithelial-specific *Nr1i2* knockout (*Nr1i2^ΔIEC^*) mice did not reveal any difference in the health monitoring after LPS treatment compared to *Nr1i2^fl/fl^* mice (76). In addition, the length of the small intestine and colon were similar between *Nr1i2^ΔIEC^* and *Nr1i2^fl/fl^* mice under LPS treatment (76).

Histological assessment of the colon after *Tcd A/B* revealed higher total damage scores (epithelial damage, the inflammatory response, and architecture damage) in the colon of *Nr1i2^−/−^* mice compared with *Nr1i2^+/+^* mice (88). The colonic sections from *Nr1i2^−/−^* mice exposed to *Tcd A/B* showed increased damage to the surface epithelium at the luminal interface, prominent patches of increased inflammatory cell infiltration in the mucosa, and increased edema underneath the muscularis mucosa that was also clearly populated with granulocytic infiltrating cells (88). Moreover, in a 3-deoxy-D-manno-octulosonic acid (KDO2)-lipid A treatment, although, there was no overt intestinal histologic evidence of inflammation in both *Nr1i2^−/−^* and *Nr1i2^+/+^* mice, FITC-dextran intestinal integrity assay showed higher permeability in *Nr1i2^−/−^* mice (36).

Colonic production of several cytokines and chemokines (*Csf2, Csf3, Cxcl1, Cxcl2*, and *Il6*) was also significantly higher in *Nr1i2^−/−^* compared with *Nr1i2^+/+^* mice under *Tcd A/B*. The expression of *Tlr4* in the colon of *Nr1i2^−/−^* mice was also increased (88). Similar higher pro-inflammatory cytokines and chemokines were also observed in *Nr1i2^−/−^* serum mice with *Listeria monocytogenes* (*Lm*) infection (89). In addition, LPS stimulation of primary intestinal myofibroblasts from the colon of *Nr1i2^+/+^* and *Nr1i2^−/−^* mice showed increased expression and concentration of various cytokines/chemokines, (*Csf2, Csf3, Cxcl1, Cxcl2*, and *Il6*) but these responses were exaggerated in *Nr1i2^−/−^* fibroblasts (38). However, no difference in inflammatory, antimicrobial, tight- and adherens-junction, proliferation gene expression in the small intestine, colon, and liver was observed between *Nr1i2^ΔIEC^* and *Nr1i2^fl/fl^* mice (76). Unusually, the jejunum of *Nr1i2^ΔIEC^* mice was characterized by a significant reduction of inflammatory (*Tlr4, Il10*, and *Tnf*), antimicrobial (*Muc2*), and tight-junction (*Tjp1* and *Ocln*) markers (76). Taken together these findings demonstrate that the lack of intestinal Nr1i2 does not aggravate LPS-induced inflammation in mice (76).

Furthermore, in primary hepatocyte cultures (PCH) the fold-induction of *Il6, Tnf*, and *Il1rn* after LPS treatment was less robust in the *Nr1i2^−/−^* cultures when compared with *Nr1i2^+/+^*, and LPS-inducible *Il1b* expression levels and Il1rn protein were enhanced in *Nr1i2^−/−^* PCHs (86). Otherwise, the *Nfkb1* liver mRNA levels expression was more important in *Nr1i2^−/−^* endotoxin-treated mice compared with *Nr1i2^+/+^*, while *Il1b* was reduced. However, after LPS treatment, no difference in inflammatory gene expression was observed in the liver between *Nr1i2^ΔIEC^* and *Nr1i2^fl/fl^* mice (76) and even in serum between *Nr1i2^−/−^* and *Nr1i2^+/+^* mice (80).

In addition to the increased inflammatory response, *Nr1i2^−/−^* mice were highly susceptible to systemic bacterial infection. When comparing colonic neutrophils isolated from *Nr1i2^+/+^* and *Nr1i2^−/−^* mice following *Tcd A/B* exposure, significant differences in cytokine expression including increased expression of Csf2, and reduced expression of Csf3 and Tnf in *Nr1i2^−/−^* neutrophils. There was no difference in the expression of Il6 between *Nr1i2^+/+^* and *Nr1i2^−/−^* neutrophils (88). In contrast, there was a significant upregulation in the expression of Il6 and Csf3 in *Nr1i2^−/−^* eosinophils, while *Nr1i2^−/−^* eosinophils also had decreased expression of Tnf *(*88*)*. Moreover, Ly6Chi monocyte infiltration into the colon was increased following *Tcd A/B* exposure, an effect that was exaggerated in *Nr1i2^−/−^* mice (88). However, both the frequency and the number of inflammatory Ly6Chi monocytes were markedly reduced in the blood, spleen, and liver of *Nr1i2^−/−^* mice after *Lm* infection (89). The reduction in inflammatory monocytes was due to accelerated monocytes cell death. Indeed, monocytes’ early apoptotic cells, late apoptotic cells, and necrotic cells were all significantly higher in *Nr1i2^−/−^* mice post-infection. In addition, although the percentage of monocytes that produced Tnf was comparable between *Nr1i2^−/−^* and *Nr1i2^+/+^* mice, *Nr1i2^−/−^* inflammatory monocytes produced significantly higher levels of Tnf and Ros1 (89). The excessive Ros1 production was indeed responsible for the increased monocyte cell death in *Nr1i2^−/−^* mice. Finally, the bacterial load at 24 hou*rs* post-Lm infection was comparable in the spleen and liver of *Nr1i2^−/−^* and *Nr1i2^+/+^* mice. However, at 48 and 72 hours after infection the bacterial burden was significantly higher in *Nr1i2^−/−^* mice in both spleen and liver (89).

In summary, whole-body *Nr1i2* knockout (*Nr1i2^−/−^*) mice but not tissue-specific *Nr1i2* knockout (*Nr1i2^ΔIEC^*) mice showed exaggerated intestinal injury under bacterial toxins induced inflammation characterized by histological damages, barrier integrity disorder, higher inflammation markers and innate immune cell infiltration and which causes early death.

#### 3.3.2 Chemicals induction

Dextran sulfate sodium (DSS) administered to *Nr1i2^+/+^* and *Nr1i2^−/−^* mice induce severe experimental colitis/IBD-like disease but without any difference in the severity of major clinical symptoms (body weight loss, rectal bleeding diarrhea, and shortening of the colon) between *Nr1i2^+/+^* and *Nr1i2^−/−^* mice (38, 81). A similar observation has been demonstrated in siRNA whole-body reduced *Nr1i2* (*Nr1i2^-KD^*) mice, where reduced Nr1i2 slightly aggravated the severity of DSS-induced IBD clinical symptoms (70). In addition, histopathology of the colon illustrated significant inflammatory infiltrates regardless of the presence of Nr1i2 (81, 90). Moreover, a significant and similar induction of all pro-inflammatory NF-κB target genes (*Nos2, Ccr2, Icam1, Ccl2, Tnf, Ifna, Il10, Il6*, and *Il1b*) mRNA mediators was observed in *Nr1i2^+/+^* and *Nr1i2^−/−^* mice after DSS treatment (91). Furthermore, mice treated with azoxymethane (AOM)/dextran sulfate sodium (DSS) induced colon cancer produced similar total tumor numbers per colon in both mice groups (92).

The absence of Nr1i2 did not increase the susceptibility of mice to DSS-induced injury. However, during healing after stopping DSS treatment, once mice began to regain body weight, *Nr1i2^+/+^* mice gained significantly more weight than *Nr1i2^−/−^* mice, suggesting a poorer recovery in the absence of the Nr1i2 (38). Moreover, macroscopic examination of the colon in these mice after healing did not reveal a difference in colon length between *Nr1i2^+/+^* and *Nr1i2^−/−^* mice; however, there was significantly greater colonic thickening in *Nr1i2^−/−^* mice. Masson’s trichrome-stained colonic sections from each strain of mice showed that the fibrotic layer of connective tissue underlying the mucosa occupied significantly more area and was significantly thicker in *Nr1i2^−/−^* mice than in *Nr1i2^+/+^* (38). In addition, the protein expression and serum concentration of immune mediators (Csf2, Csf3, Tnf, Il5, Cxcl2, and Cxcl9) showed greater levels in *Nr1i2^−/−^* mice serum (38).

Furthermore, intestinal fibroblast-specific *Nr1i2* knockout (*Nr1i2^ΔCol1a2^*) mice revealed the same phenotype of increased inflammation and fibrosis observed in *Nr1i2^−/−^* mice (38). The colon of *Nr1i2^ΔCol1a2^* mice was significantly thicker than *Nr1i2^fl/fl^* control mice, and histological examination of colon sections showed significantly larger areas of collagen deposition in *Nr1i2^ΔCol1a2^* mice. In addition, the expression of collagen (*Col1a1, Col1a2*, and *Col3a1*), profibrotic collagenases (*Mmp3, Mmp8*, and *Mmp9*) and cytokines (*Il1b, Il18, Csf2, Csf3, Cxcl1*, and *Cxcl2*) were significantly higher in the healing colon of *Nr1i2^ΔCol1a2^* than in counterpart *Nr1i2^fl/fl^* mice (38). However, the expression of neutrophil profibrotic antimicrobial enzymes (*Elane, Cybb, Nox1, Mpo*) was similar between *Nr1i2^ΔCol1a2^* and *Nr1i2^fl/fl^* mice (38). Furthermore, all these prior healing histological and mRNA expression differences after DSS treatment were not observed in intestinal epithelial-specific *Nr1i2* knockout (*Nr1i2^ΔIEC^*) compared to *Nr1i2^fl/fl^* mice (38).

Other chemicals demonstrate a completely different effect. Polychlorinated biphenyls (PCB-153) chronic exposure treatment showed premature death in male *Nr1i2^−/−^* mice (93). A similar result was shown in the anti-CD3 antibody-induced intestinal inflammation-edema model. *Nr1i2^−/−^* mice succumbed to intestinal edema and died earlier. In addition, there was near-complete blunting of crypt-villus architecture in *Nr1i2^−/−^* (36). Moreover, in the indomethacin-induced small intestine inflammation model, *Nr1i2^−/−^* mice showed more inflammation by significantly increased histologic damage score and enhanced basal MPO activity compared to *Nr1i2^+/+^* mice (36). In addition, cytomix (combination of Tnf, Il1b, and Ifnγ) treatment increased expression of various cytokines/chemokines and these were exaggerated in *Nr1i2^−/−^* primary intestinal myofibroblasts, except Il6 production that was not different between strains (38). Furthermore, gut microbiome comparison between *Nr1i2^−/−^ vs. Nr1i2^+/+^* and PCB *vs.* vehicle conditions, showed that 9 taxa were commonly regulated, among which a member of the *Ruminococcaceae* family was consistently suppressed by Nr1i2 under PCB-exposed conditions, whereas *Streptococcus* sp. *and Anaeroplasma* sp. were consistently induced by Nr1i2 under PCB-exposed conditions (84). Finally, acetaminophen (APAP) administration caused a time-dependent elevation of serum ALT levels and histological liver injury that were more severe in *Nr1i2^−/−^* mice (94). Finally, Lithocholic acid (LCA) treatment showed massive histological damage in the livers of *Nr1i2^−/−^* compared with *Nr1i2^+/+^* mice with a high ALT serum level and the extensive inflammatory and neutrophil infiltration prominent in the necrotic lesions and surrounding areas (67, 95). Conversely, silencing Nr1i2 expression using siRNA slightly ameliorated the pathological changes in the liver and decreased serum elevation of ALT, AST, and ALP activity in *Nr1i2^-KD^* (71). However, no kidney morphological differences were identified between *Nr1i2^−/−^* and *Nr1i2^+/+^* mice under APAP treatment (94).

To summarize, whole-body *Nr1i2* knockout (*Nr1i2*^−/−^) mice and intestinal fibroblast-specific *Nr1i2* knockout (*Nr1i2^ΔCol1a2^*) but not epithelial-specific *Nr1i2* knockout (*Nr1i2^ΔIEC^*) mice showed similar intestinal injury compared to their control under DSS-induced colitis. However, during healing, both *Nr1i2*^−/−^ and *Nr1i2^ΔCol1a2^* mice showed poorer recovery. These findings identify mesenchymal Nr1i2 as a critical regulator of post-inflammatory tissue repair. Furthermore, other chemicals demonstrate a more drastic effect in *Nr1i2*^−/−^ mice compared to *Nr1i2*^+/+^.

#### 3.3.3 Experimental injury induction

In the experimental necrotizing entero-colitis (NEC), gross inspection and histopathological assessment exhibited a remarkably heightened gut injury in the *Nr1i2^−/−^* mice (28). In addition, in the gastrointestinal ischemia-reperfusion model, the change in permeability as assessed by a change in recovery of mean levels of FITC-dextran in the serum of mice was higher in *Nr1i2^−/−^* mice than *Nr1i2^+/+^* (36). Moreover, experimental NEC induced more markers of intestinal barrier function disturbance in *Nr1i2^−/−^* mice compared to mice *Nr1i2^+/+^*. The Gja1 dramatically increased in *Nr1i2^−/−^* mice subjected to experimental NEC, which could indicate gap-junction disassembly with an accumulation of dysfunctional isoforms (28). Experimental NEC resulted also in reduced levels of Muc2 in *Nr1i2^−/−^* mice. Additionally, experimental NEC also induced a profound increase in transcripts for the pro-inflammatory cytokine *Il6, and Tlr4* expression, which was greater in intestinal tissues obtained from *Nr1i2^−/−^* mice (28).

However, hemorrhagic shock (HS/R) showed attenuated hepatic injury in *Nr1i2^−/−^* mice (96). In addition, spinal cord injury (SCI) showed by immuno-histochemical staining that a microglia marker (F4/80) and Nlrp3 expression were significantly decreased in *Nr1i2^−/−^* mice (87). TUNEL staining showed significantly alleviated apoptosis in the *Nr1i2^−/−^* group (87). Examined the activities of antioxidant enzymes, superoxide dismutase (Sod) and glutathione peroxidase (Gpx), and the expression of markers of oxidative stress (Mda, 4-Hne, and 8-Oxo) showed a significant decrease of Mda, 4-Hne, and 8-Oxo content and increase activity of Sod and Gpx in *Nr1i2^−/−^* mice spinal tissues (87). Taken together, these results suggested that deletion of the *Nr1i2* gene promoted the recovery of motor function and alleviated apoptosis, inflammation, and oxidative stress (87).

In conclusion, whole-body *Nr1i2* knockout (*Nr1i2*^−/−^) mice showed exaggerated intestinal inflammation under experimental injury with elevated barrier integrity disorder, higher inflammation markers, and reduced junctions gene expression. Conversely, mice showed alleviated liver and spinal injury.

#### 3.3.4 Diet induction

High-fat diet (HFD) experiments showed that the absence of *Nr1i2* (*Nr1i2^−/−^* mice) attenuated HFD-induced hepatic steatosis, hepatic inflammatory cell infiltration, and decreased mRNA levels of pro-inflammatory (*Tnf* and *Il6*) (97–99). Moreover, under HFD and compared to *Nr1i2^+/+^* mice, *Nr1i2^−/−^* did not show any up-regulation of pro-inflammation related genes (*Cd74, Il2rg*, and *Clec7a*), down-regulation of genes encoding tight junctions (*Cldn5*), up-regulation of genes involved in microbial response (*Ly96* and *App*), up-regulation biomarkers of oxidative stress (*Nfe2I2, Gpx4*, and *Casp3*), as well as cell proliferation/cancer-related genes (*Lgals1, Mdm2, Mapk3, Ptpra, Pea15a*, and *Tgfb1*) (99). Furthermore, *Nr1i2^−/−^* gut microbiome did not exhibit down-regulation of the anti-inflammatory *Bifidobacterium* genus, up-regulation of the pro-inflammatory Lactobacillus genus, down-regulation anti-obesity marker *Allobaculum* (99).

In conclusion, whole-body *Nr1i2* knockout (*Nr1i2*^−/−^) mice showed attenuated exaggerated inflammation under diet disturbance with reduced hepatic inflammation, and absence of gut dysbiosis.

### 3.4 *Nr1i2* ligands treatment under inflammatory conditions

Given that Nr1i2 deficiency exacerbates inflammatory and intestinal injury in response to inflammatory stimuli, while its expression demonstrates an anti-inflammatory role, the investigation into Nr1i2 ligand treatment under such inflammatory conditions became evident and would reveal that activating Nr1i2 promotes protective or restorative effects.

#### 3.4.1 Under bacterial toxins-induced inflammation

Histological assessment of the colon from *Tcd A/B*‐exposed mice showed reduced damage to the colon with only minor epithelial damage, reduced percentage and a number of infiltrating neutrophils into the colonic *lamina propria*, and minimal immune cell influx into the mucosa with limited edema in the layer underlying the muscularis mucosae in mice treated with PCN (88). These changes were reflected in the decreased total damage score in *Nr1i2^+/+^* PCN‐treated mice exposed to *Tcd A/B* when compared to vehicle‐treated, *Tcd A/B*‐exposed mice (88). Further, when compared to vehicle‐treated mice, PCN reduced colonic expression of Cxcl1, Cxcl2, Cxcl10, Csf2, Tnf, Il6, and Il17a following *Tcd A/B* treatment (88). Moreover, pretreating mice with PCN attenuated KDO2-induced barrier disruption in *Nr1i2^+/+^* mice, but not *Nr1i2^−/−^* mice (100). Likewise, Indole-3-Propionic Acid (IPA) treatment notably decreased *Tnf* mRNA expression induced by KDO2 more in the *Nr1i2^+/+^* mice intestinal epithelium relative to *Nr1i2^−/−^* mice (36). Furthermore, LCA treatment of *Nr1i2^+/+^* intestinal organoids reduced LPS-induced transcription of *Il6* but did not alter it in intestinal organoids from *Nr1i2^−/−^* mice and did not alter LPS-induced transcription of *Tnf* even in *Nr1i2^+/+^* intestinal organoids (28).

Otherwise, in *Nr1i2^+/+^* PCHs, when compared with LPS treatment alone, treatment with PCN and subsequent cotreatment together with LPS produced significantly lower expression levels of several notable LPS-inducible NF-κB target genes, including *Il1b, Il6, Tnf*, and *Il1rn*. Contrary to the results obtained with *Nr1i2^+/+^*, pretreatment of *Nr1i2^−/−^* PCHs with PCN and subsequently with LPS did not suppress subsequent LPS-inducible expression of any of the NF-κB target genes (86). However, in *Nr1i2^+/+^* LPS-primed mouse macrophages, treatment with PCN was able to trigger the release of Il1b, while, LPS-primed peritoneal macrophages isolated from *Nr1i2^−/−^* mice did not exhibit Casp1 activation nor secrete Il1b in response to PCN (101).

In summary, *Nr1i2^+/+^* mice and cells but not whole-body *Nr1i2* knockout (*Nr1i2*^−/−^) showed a restorative effect of *Nr1i2* agonists treatment following bacterial toxin-induced inflammation.

#### 3.4.2 Under chemical-induced inflammation

*Nr1i2^+/+^* mice treated with PCN demonstrated a decrease in the severity of DSS-induced IBD compared with vehicle-treated mice. PCN significantly improved body weight loss, diarrhea scores, bleeding scores, and colon lengths (70, 81, 102). Histological analysis showed that DSS-induced colonic injury was less severe in PCN-treated mice (81, 100, 102). PCN treatment also significantly decreased gross colon thickness, decreased fibrotic area and thickness in the submucosal area, and decreased necrotic foci when compared with vehicle-treated mice (38, 70, 100). However, although a noted tendency, PCN treatment did not significantly decrease the number of apoptotic cells (102). Moreover, PCN did not improve the clinical symptoms and histological damages of DSS-induced IBD in *Nr1i2^−/−^* mice (38, 81, 102). The protective effects of PCN in DSS-induced IBD were mediated via an Nr1i2-dependent mechanism. PCN treatment decreased the DSS induction of NF-κB target genes (*Il1b, Il10, Tnf, Ccr2*, and *Nos2*) (70, 81, 102). In addition, PCN administration decreased expression of collagen (*Col1a1, Col1a2*, and *Col3a1*), of colony-stimulating factor (*Csf2* and *Csf3*) and the neutrophil chemokines C-X-C Motif chemokine ligand (*Cxcl1* and *Cxcl2*) following DSS treatment only in *Nr1i2^+/+^* mice (38). However, PCN treatment decreased DSS-induced expression of *Il6* and *Ccl2* mRNA in both *Nr1i2^+/+^* and *Nr1i2^−/−^* mice, suggesting that PCN may act independently of Nr1i2 on specific NF-κB target genes (81).

Comparable clinical, histological, and inflammatory markers results were demonstrated after treating DSS-induced IBD mice with herbal compounds Tanshinone IIA (Tan IIA) (70), St. John’s Wort (SJW) (69), Baicalein and its O-glucuronide Baicalin (90) or with microbiota compound IPA (38) and Felix Kopp Kortagere 6 (FKK6) (37). This protective effect was even observed in the small intestine of DSS-induced IBD mice treated with PCN or SJW (69). Like the PCN, none of these herbal and bacterial compound treatments improved the damages of DSS-induced IBD in *Nr1i2^−/−^* mice, demonstrating a Nr1i2-dependent effect. However, mice with reduced *Nr1i2* expression slightly aggravated the severity of DSS-induced IBD and this damage was slightly augmented by treatment with Tan IIA or PCN. Levels of inflammatory mediators were lower in siRNA *Nr1i2*-silenced (*Nr1i2^-KD^*) mice treated with Tan IIA or PCN, and the effects of treatment were markedly decreased (70).

Otherwise, intestinal commensal-depleted *Nr1i2^+/+^* and *Nr1i2^−/−^* mice were exposed to live or heat-killed *C. sporogenes* and subsequently exposed to indomethacin. Only live *C. sporogenes*, but not the heat-killed bacterial inoculation, led to the production of IPA *in vivo*. There was a significant reduction in intestinal histologic injury and mucosal MPO enzyme activity in *Nr1i2^+/+^* mice only. In addition, IPA direct administration followed by indomethacin significantly reduced FITC-dextran permeability in *Nr1i2^+/+^* but not in *Nr1i2^−/−^* mice (36). Likewise, both SJW and its major component hyperforin extract significantly attenuated *Tnf*-induced NF-κB activation in primary intestinal organoids isolated from the mice distal ileum but not in primary intestinal organoids from *Nr1i2^−/−^* mice (103). In addition, *Tnf*-induced NF-κB was significantly inhibited by PCN treatment in hepatocytes isolated *Nr1i2^+/+^* mice only (79). Moreover, treatment with PCN dramatically reduced the detrimental effects of LCA on the livers of *Nr1i2^+/+^* mice (67, 95). In marked contrast, treatment with PCN did not reverse the hepatotoxicity of LCA in the *Nr1i2^−/−^* mice (67, 95). A significant loss of PCN-mediated xenoprotection in *Nr1i2^−/−^* mice was also observed when the whole-body knockout mice were challenged with two other xeno-toxicants, the anesthetic Tribromoethanol and the muscle relaxant Zoxazolamine (95). In addition, siRNA *Nr1i2*-silenced (*Nr1i2^-KD^*) and *Nr1i2^-WT^* mice that were treated with Tan IIA or PCN exhibited remarkably fewer sub-capsular foci of white discolorations in the liver compared with LCA-treated mice. *Nr1i2^-KD^* and *Nr1i2^-WT^* mice that were treated with Tan IIA exhibited further decreased ALT, AST, and ALP (71). Furthermore, compared with tetrachloromethane (CCl4)-treated *Nr1i2^-WT^* mice, liver histological observation and changes of AST and ALT showed that Ginkgolide A exhibited a preventive effect against CCl4-induced liver necrosis. Feeding Ginkgolide A showed no obvious curative effect on CCl4-induced hepatitis in shRNA *Nr1i2*-silenced (*Nr1i2^-KD^*) mice (69). However, APAP dramatically enhanced ALT levels in *Nr1i2^+/+^* mice following treatment with PCN. In contrast, following the APAP administration, the *Nr1i2^−/−^* mice pretreated with PCN exhibited reduced ALT levels compared to those pretreated with vehicles. In addition, treatment with PCN dramatically enhanced the acetaminophen-induced hepatic injury in *Nr1i2^+/+^* mice only (94). Finally, treatment with PCN markedly preserved the normal morphology of the kidney and significantly attenuated the proximal tubule damage induced by Cisplatin (CIS). However, PCN treatment failed to confer protection against cisplatin-induced renal injury in *Nr1i2^−/−^* mice (68).

In conclusion, many studies have extensively addressed the effect of Nr1i2 ligand treatments under chemical-induced inflammation using whole-body *Nr1i2* knockout or knockdown. *Nr1i2* preactivation showed a protective effect in control mice. This effect was reduced in the knockdown (*Nr1i2^-KD^*) mice and absent in the whole-body knockout (*Nr1i2^−/−^*) mice.

#### 3.4.3 Under experimental injury or diet-induced inflammation

Under experimental NEC, in the early stage, despite the non-difference in histologic grading of NEC severity, the transcription of *Il6* and *Tlr4* in the terminal ileum of only *Nr1i2^+/+^* mice receiving LCA was reduced compared to mice not given LCA, indicating that LCA protected against the upregulation of pro-inflammatory markers but failed in *Nr1i2^−/−^* mice (28). However, as NEC progressed to a late stage, the levels of transcripts for *Il6* and *Tlr4* were comparable between LCA and non-LCA-fed *Nr1i2^+/+^* mice (28). Furthermore, treatment with PCN sensitized *Nr1i2^+/+^* mice to HS-induced hepatic injury by increasing necrotic area, Suzuki scores, and the serum ALT level (96). A similar pattern of Nr1i2-dependent sensitization was observed in mice pre-treated with dexamethasone (DEX), while the sensitizing effect of PCN and DEX was abolished in *Nr1i2^−/−^* mice. (96). Finally, HFD increased plasma ALT levels irrespective of the Nr1i2 expression, and PCN treatment further elevated ALT in the *Nr1i2^+/+^* mice (98).

To summarize, few studies have explored the effect of Nr1i2 ligand treatments under experimental injury or diet-induced inflammation. Nr1i2 activation showed a controversial effect in *Nr1i2^+/+^* mice and no effect in the *Nr1i2^−/−^* mice.

## 4 Discussion

**Figure 1:**
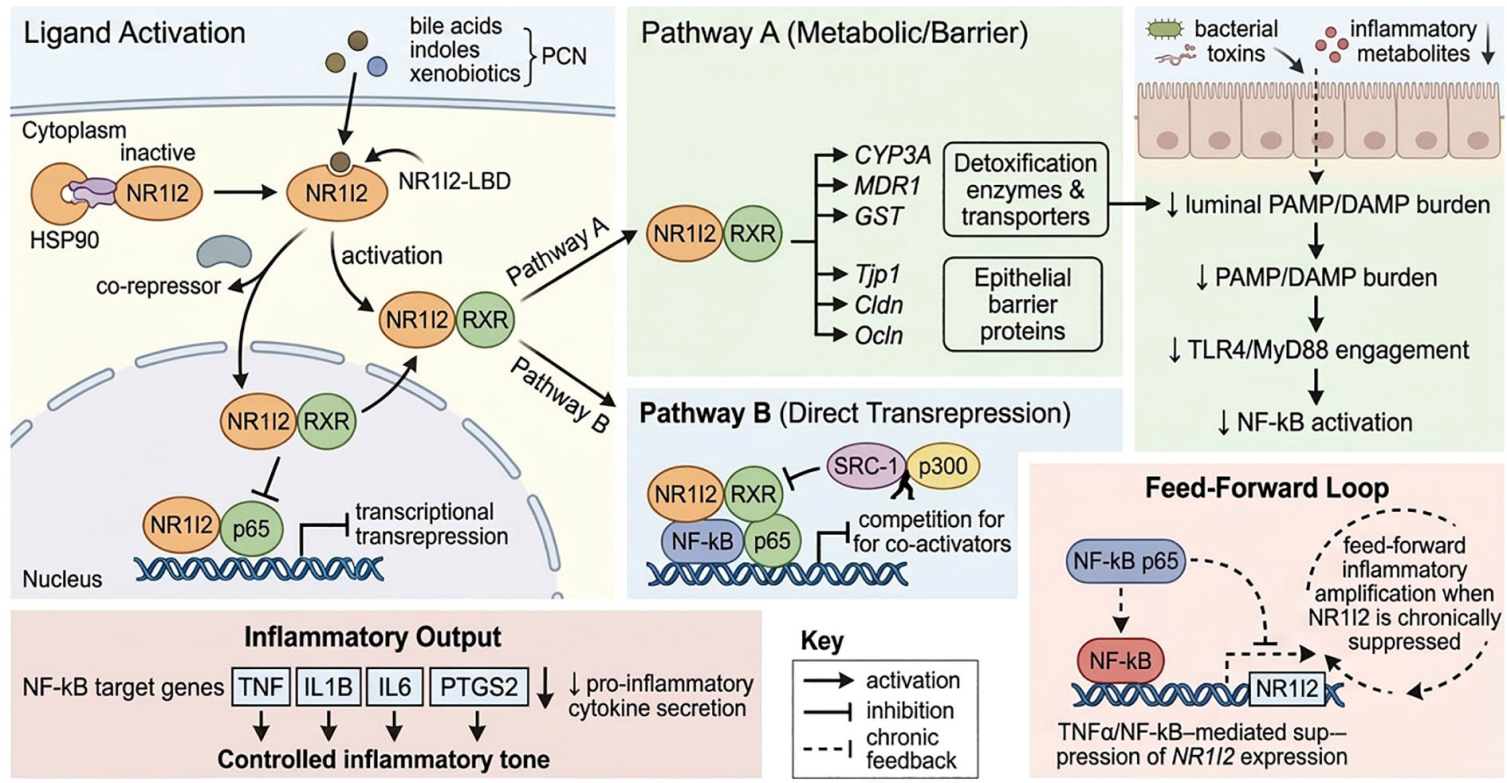
NR1I2 as a gatekeeper of innate inflammatory signaling. Upon ligand binding (bile acids, microbial indoles, xenobiotics), NR1I2 dissociates from cytoplasmic co-repressors, translocates to the nucleus, and heterodimerizes with RXR to activate target gene transcription. NR1I2 restrains innate inflammatory signaling through two parallel pathways. Pathway A (Metabolic/Barrier): NR1I2 induces phase I/II metabolic enzymes (*CYP3A*, *GST*), drug transporters (*MDR1*), and epithelial barrier components, collectively reducing the mucosal burden of pathogen-associated molecular patterns (PAMPs) and damage-associated molecular patterns (DAMPs). This diminished ligand availability limits TLR4/MyD88 engagement and subsequent NF-κB activation. Pathway B (Direct Transrepression): NR1I2 directly inhibits NF-κB–dependent transcription through competition for shared co-activators and reciprocal transrepression mechanisms. Together, these pathways reduce expression of NF-κB target genes (*TNF*, *IL1B*, *IL6*, *PTGS2*) and maintain a controlled inflammatory tone. Under chronic inflammatory conditions (dashed inhibitory line), sustained NF-κB activity suppresses *NR1I2* gene expression, creating a feed-forward loop that impairs detoxification and barrier integrity, further amplifying innate immune activation. Solid arrows indicate activation; blunt-ended lines indicate inhibition; dashed lines indicate chronic feedback suppression. The conceptual design of this figure was assisted by generative AI tools.

**Figure 2:**
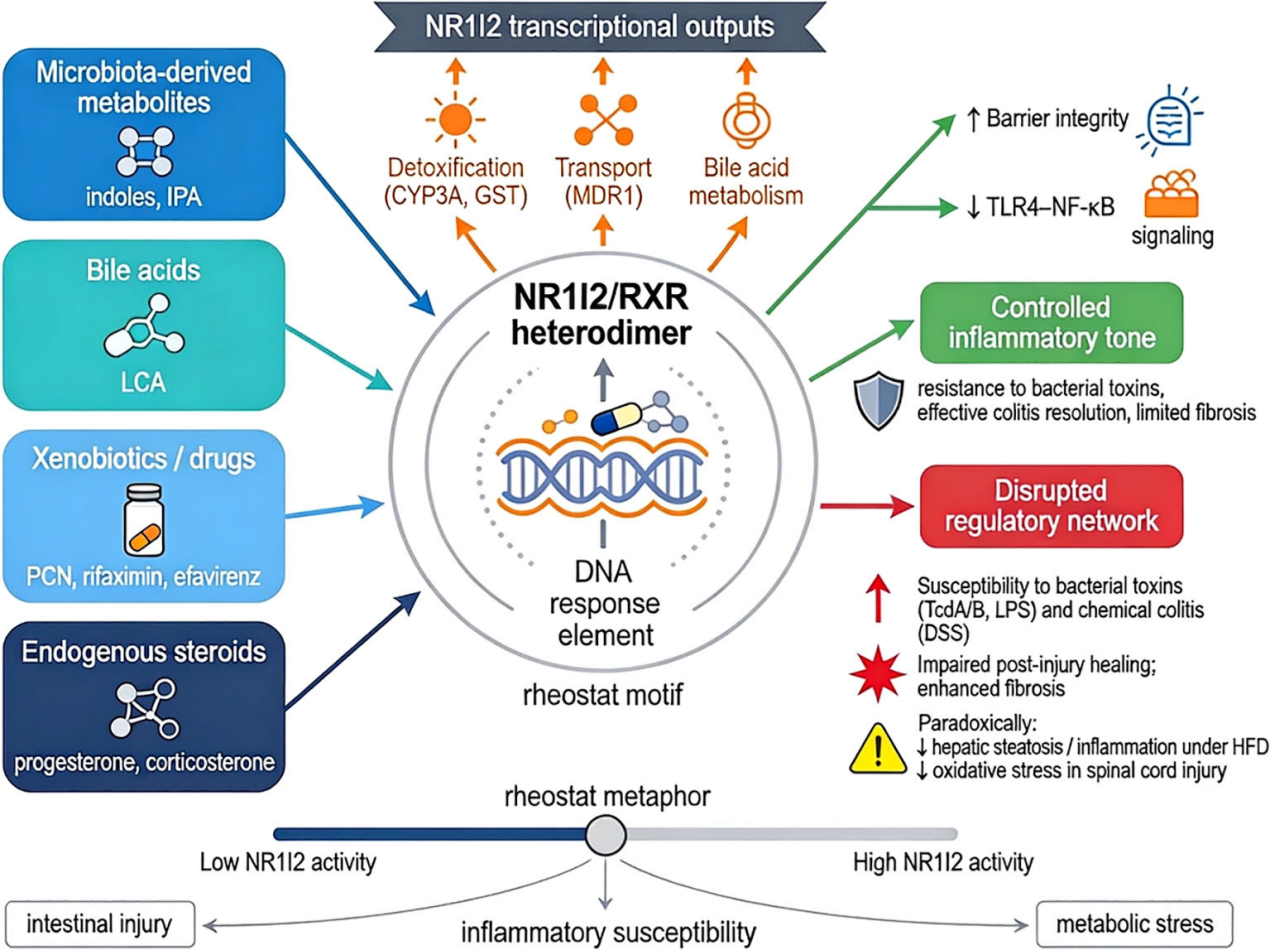
NR1I2 as an immune–metabolic rheostat. NR1I2 senses a diverse array of metabolic and environmental signals, including microbiota-derived tryptophan metabolites (e.g., indole-3-propionic acid), secondary bile acids (e.g., lithocholic acid), xenobiotics, and pharmacological agents, and in response induces transcriptional programs for detoxification (CYP3A, GST), transmembrane transport (MDR1), and bile acid metabolism. These programs collectively support epithelial barrier integrity and restrain TLR4–NF-κB–mediated inflammatory signaling. With functional NR1I2, the host maintains controlled inflammatory responses: resistance to bacterial toxin–induced injury, effective resolution of chemical colitis, and limited post-inflammatory fibrosis. In the absence of NR1I2, this regulatory network is disrupted, resulting in context-dependent phenotypes: (1) heightened susceptibility to bacterial toxins (TcdA/B, LPS) and chemical colitis (DSS); (2) impaired tissue repair with enhanced fibrosis following intestinal injury; yet paradoxically, (3) attenuated hepatic steatosis and inflammation under high-fat diet and reduced oxidative stress following spinal cord injury. This context-dependency underscores that NR1I2 functions as a rheostat, calibrating inflammatory tone according to the metabolic and environmental landscape, rather than as a binary anti-inflammatory switch. The conceptual design of this figure was assisted by generative AI tools.

This review highlights a central concept emerging from studies using *Nr1i2*-deficient mouse models: the nuclear receptor gene *Nr1i2* and its protein product Nr1i2 regulate inflammation in a highly context-dependent manner. Its functional impact is shaped by multiple variables, including the genetic strategy employed, the cellular compartment affected, the nature and timing of inflammatory stimuli, and the metabolic and microbial environment (38, 66, 67, 81). Collectively, the available data support the view that Nr1i2 is not a simple anti-inflammatory switch, but rather an integrative immune–metabolic regulator positioned at the interface of xenobiotic sensing, epithelial barrier function, innate immune signaling, and host–microbiota interactions (36, 84). Consistent with this framework, *Nr1i2* deficiency can either exacerbate or attenuate inflammatory responses depending on the dominant tissue stressor and compartment-specific signaling environment. This integrative regulatory concept is summarized in **Figures 1**, **2**.

An important and recurrent feature of the literature summarized in this review is the apparent heterogeneity in phenotypes observed across *Nr1i2*-deficient mouse models. These differences include divergent basal expression of detoxification genes among whole-body knockout lines (66, 67), variability in microbiota composition (79, 84), and marked differences between whole-body and tissue-specific deletions (38, 73, 76). Rather than reflecting experimental inconsistency, these findings reveal that Nr1i2 functions within a distributed regulatory network whose impact depends on allelic design, cellular compartment, and environmental exposure. The phenotypic differences and inflammatory susceptibilities across models are summarized in **Supplementary Table 1**.

Several major determinants help explain these context-dependent outcomes. First, allelic targeting strategies differ between knockout models, potentially affecting residual transcription, developmental compensation, or secondary activation of related nuclear receptors such as the constitutive androstane receptor (Nr1i3/Car) or farnesoid X receptor (Nr1h4/Fxr) (19, 66, 67). Second, compartment-specific functional dominance plays a critical role. Whole-body deletion disrupts coordinated Nr1i2 activity across epithelial, mesenchymal, immune, and hepatic compartments, whereas epithelial-restricted deletion leaves systemic detoxification and immune regulation largely intact (38, 73, 76). Third, microbiota composition strongly influences inflammatory tone, as Nr1i2 regulates host–microbe metabolic interactions in an age-, sex-, and environment-dependent manner (82, 84). Finally, the nature of inflammatory stress is a key determinant. *Nr1i2* deficiency increases susceptibility to bacterial toxins, experimental injury, and xenobiotic stress, but can attenuate inflammatory responses under selected metabolic conditions such as high-fat diet (28, 36, 97–99). Together, these factors provide a unifying explanation for model-specific phenotypes.

**Figure 3:**
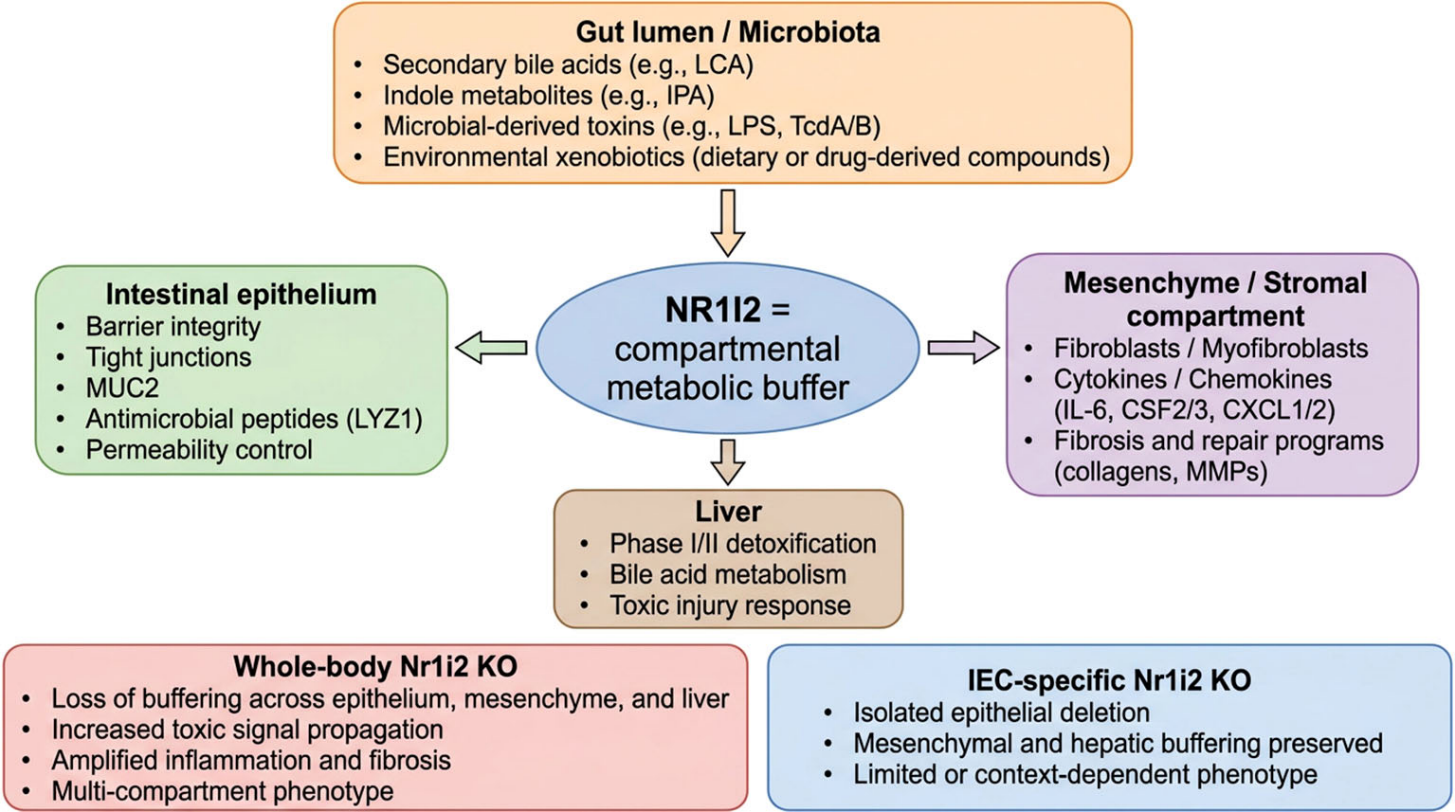
NR1I2 as a compartmental metabolic buffer along the gut–liver axis. Signals arising from the gut lumen and microbiota, including secondary bile acids (e.g., LCA), indole metabolites (e.g., IPA), microbial toxins (e.g., LPS and TcdA/B), and dietary or drug-derived xenobiotics, converge on NR1I2. NR1I2 functions as a metabolic buffer across multiple compartments, including intestinal epithelium, mesenchymal/stromal cells, and liver, where it supports barrier integrity, tight junctions, mucin and antimicrobial peptide production, detoxification pathways, bile acid metabolism, and responses to toxic injury. Whole-body loss of Nr1i2 disrupts buffering in these compartments, enhancing toxic signal propagation, inflammation, fibrosis, and a multicompartment disease phenotype, whereas, for example, IEC-specific Nr1i2 deletion primarily affects epithelial signaling with more limited or context-dependent consequences. The conceptual design of this figure was assisted by generative AI tools.

A central insight emerging from this review is that epithelial-specific *Nr1i2* deletion alone is frequently insufficient to reproduce the inflammatory phenotypes observed in whole-body knockout mice. Intestinal epithelial-specific *Nr1i2* knockout mice maintain normal barrier integrity and inflammatory profiles under basal and inflammatory conditions (76), whereas whole-body *Nr1i2*^−/−^ mice exhibit impaired barrier function, increased inflammatory signaling, and heightened susceptibility to injury (36). This difference likely reflects the distributed nature of Nr1i2 function. fibroblast-specific *Nr1i2* deletion reproduces the impaired repair and fibrosis phenotype observed in whole-body knockout mice, highlighting a critical stromal contribution (38). Additionally, Nr1i2 ligands and substrates circulate systemically, and whole-body deletion disrupts detoxification and metabolic homeostasis across multiple organs (67, 94). These findings demonstrate that the Nr1i2 protein functions as a multi-compartmental regulator of inflammatory homeostasis, as illustrated in **Figure 3**; **Supplementary Table 1**.

Mechanistically, the Nr1i2 protein restrains inflammatory activation through complementary direct and indirect pathways (**Figure 1**). At the transcriptional level, Nr1i2 directly interacts with inflammatory signaling through reciprocal repression of *Nfkb1* activity. Activation of Nr1i2 inhibits NF-κB-dependent transcription through co-regulator competition and transcriptional repression, whereas inflammatory cytokines such as Tnf suppress *Nr1i2* gene expression through NF-κB-dependent mechanisms (60, 61, 79). This reciprocal relationship establishes a feed-forward loop that amplifies inflammation when Nr1i2 expression is reduced.

In parallel, Nr1i2 exerts indirect anti-inflammatory effects by regulating detoxification pathways and maintaining epithelial barrier integrity. Activation of Nr1i2 induces enzymes and transporters involved in xenobiotic metabolism and bile acid handling, thereby reducing epithelial exposure to microbial metabolites and toxic ligands capable of activating innate immune receptors such as Tlr4 (26–28, 36). In this framework, the Nr1i2 protein functions as an upstream regulator of inflammatory tone by controlling the availability of innate immune stimuli. Loss of Nr1i2 disrupts detoxification and barrier integrity, increasing epithelial exposure to microbial and xenobiotic ligands, enhancing Tlr4 signaling, and amplifying NF-κB-dependent inflammatory responses. This integrated immune–metabolic control explains why whole-body deletion produces broader inflammatory phenotypes than epithelial-restricted deficiency.

Importantly, Nr1i2 also plays a critical role in regulating tissue repair following inflammatory injury. Although acute DSS-induced inflammation is often similar between genotypes, Nr1i2 deficiency significantly impairs recovery and promotes fibrosis, particularly in fibroblast-specific knockout mice (38). These findings identify mesenchymal Nr1i2 as a key checkpoint controlling post-inflammatory tissue repair and fibrosis, highlighting a function distinct from its role in acute inflammatory regulation.

Nr1i2 also integrates host and microbial metabolism through responsiveness to microbiota-derived ligands such as indole metabolites and secondary bile acids (36–38). These ligands activate Nr1i2-dependent transcriptional programs that reinforce barrier function and restrain excessive immune activation. Loss of Nr1i2 disrupts this immune–metabolic equilibrium, predisposing the host to exaggerated inflammatory responses when barrier integrity is compromised.

Finally, Nr1i2 operates within a broader network of environmental sensing nuclear receptors that regulate immune and metabolic homeostasis. This network includes the constitutive androstane receptor (CAR/NR1I3), farnesoid X receptor (FXR/NR1H4), vitamin D receptor (VDR), peroxisome proliferator-activated receptors (PPARs), and the aryl hydrocarbon receptor (AHR), which collectively integrate xenobiotic, metabolic, and microbial signals (19, 34, 35). Within this network, the Nr1i2 protein plays a distinct role by coordinating detoxification programs, epithelial barrier maintenance, and innate immune signaling.

Together, these findings support a unifying model in which the *Nr1i2* gene and its protein product Nr1i2 function as an immune–metabolic rheostat that regulates inflammatory susceptibility through coordinated detoxification, microbial sensing, and innate immune restraint. This integrated mechanistic framework reconciles divergent phenotypes observed across models and provides a conceptual basis for understanding how Nr1i2 maintains inflammatory homeostasis across tissues and contexts, as summarized in **Figures 1**–**3**.

Findings from *Nr1i2*-deficient mice align closely with human IBD data. In active ulcerative colitis, *NR1I2* expression in colonic epithelium is significantly reduced, and in Crohn’s disease it is reported as either unchanged or decreased (60, 61). *NR1I2* levels in Crohn’s colonic biopsies inversely correlate with mucosal NF-κB activity and IL-8 expression (62), and inflammatory cytokines like TNFα can suppress *NR1I2* transcription via NF-κB (61). These observations mirror the reciprocal NR1I2–NF-κB repression loop seen in mice and highlight NR1I2 as an epithelial “brake” on intestinal inflammation.

Additionally, reduced Nr1i2 protein expression levels are observed in the liver in mouse models of ulcerative colitis (63, 64), and *Nr1i2* expression is downregulated in the intestine of mice treated with LPS (65) and in transgenic mice with constitutive expression of *Tnf* (61). Common *NR1I2* polymorphisms exist in humans (4), but thus far no clear association with IBD susceptibility has emerged (35).

From a therapeutic perspective, multiple lines of evidence support NR1I2 as a drug target: in mice, Nr1i2 agonists ameliorate experimental colitis by dampening NF-κB signaling (81, 102), and in patients, rifaximin, a gut-targeted NR1I2 ligand, has shown clinical efficacy in reducing IBD activity (91). However, NR1I2 activation can be double-edged. Systemic NR1I2 agonists like efavirenz induce hepatic steatosis, elevated plasma ALT and AST levels, and increased hepatic inflammation gene (*Ccl2*) expression in a NR1I2-dependent manner (72), and excessive NR1I2 activity may exacerbate acute organ injury (96). Notably, rodent-specific ligands such as PCN do not activate human NR1I2 due to species differences in the ligand-binding domain (10). These species differences mandate caution when extrapolating preclinical efficacy data to human dosing.

Taken together, the evidence supports the therapeutic rationale for carefully designed, gut-selective NR1I2 modulators in IBD, while highlighting that ligand specificity, tissue context, and species pharmacology must be integrated into any clinical development strategy.

## 5 Conclusion

Studies using genetically modified mouse models have established that the nuclear receptor gene *Nr1i2* and its protein product Nr1i2 play a central role in coordinating metabolic and inflammatory homeostasis. Rather than functioning as a simple suppressor of inflammatory pathways, Nr1i2 acts as an integrative immune–metabolic regulator that modulates inflammatory susceptibility through combined effects on xenobiotic detoxification, epithelial barrier integrity, and innate immune signaling.

Evidence from whole-body, knockdown, and tissue-specific knockout models demonstrates that the impact of *Nr1i2* deficiency is highly context-dependent and shaped by the nature of the inflammatory insult, the dominant cellular compartment involved, and the systemic metabolic environment. In particular, fibroblast-specific *Nr1i2* deficiency has revealed a critical role in regulating post-inflammatory tissue repair and fibrosis, identifying stromal Nr1i2 as an essential checkpoint during recovery from intestinal injury.

Mechanistically, Nr1i2 integrates environmental, microbial, and host-derived signals through both direct and indirect pathways. Through reciprocal interactions with NF-κB signaling and regulation of detoxification and metabolic pathways, Nr1i2 functions as an upstream regulator of inflammatory tone linking metabolic stress sensing to innate immune control.

Taken together, this body of work positions Nr1i2 not as a simple anti-inflammatory regulator, but as a context-sensitive molecular rheostat that adjusts inflammatory tone according to metabolic and environmental cues. This integrated framework reconciles previously divergent observations and establishes Nr1i2 as a key regulator of inflammatory homeostasis across tissues and contexts.

## References

[1] Kliewer SA Moore JT Wade L Staudinger JL Watson MA Jones SA . An orphan nuclear receptor activated by pregnanes defines a novel steroid signaling pathway. Cell. (1998) 92:73–82. doi: 10.1016/s0092-8674(00)80900-9. PMID: 9489701

[2] Oladimeji PO Chen T . PXR: More than just a master xenobiotic receptor. Mol Pharmacol. (2018) 93:119–27. doi: 10.1124/mol.117.110155. PMID: 29113993 PMC5767680

[3] Xing Y Yan J Niu Y . PXR: a center of transcriptional regulation in cancer. Acta Pharm Sin B. (2020) 10:197–206. doi: 10.1016/j.apsb.2019.06.012. PMID: 32082968 PMC7016272

[4] Zhang J Kuehl P Green ED Touchman JW Watkins PB Daly A . The human pregnane X receptor: genomic structure and identification and functional characterization of natural allelic variants. Pharmacogenetics. (2001) 11:555–72. doi: 10.1097/00008571-200110000-00003. PMID: 11668216

[5] Zhang B Xie W Krasowski MD . PXR: a xenobiotic receptor of diverse function implicated in pharmacogenetics. Pharmacogenomics. (2008) 9:1695–709. doi: 10.2217/14622416.9.11.1695. PMID: 19018724 PMC2593625

[6] Pavek P . Pregnane X receptor (PXR)-mediated gene repression and cross-talk of PXR with other nuclear receptors via coactivator interactions. Front Pharmacol. (2016) 7:456. doi: 10.3389/fphar.2016.00456. PMID: 27932985 PMC5122737

[7] Pavek P Dvorak Z . Xenobiotic-induced transcriptional regulation of xenobiotic metabolizing enzymes of the cytochrome P450 superfamily in human extrahepatic tissues. Curr Drug Metab. (2008) 9:129–43. doi: 10.2174/138920008783571774. PMID: 18288955

[8] Carnahan VE Redinbo MR . Structure and function of the human nuclear xenobiotic receptor PXR. Curr Drug Metab. (2005) 6:357–67. doi: 10.2174/1389200054633844. PMID: 16101574

[9] Moore DD Kato S Xie W Mangelsdorf DJ Schmidt DR Xiao R . International Union of Pharmacology. LXII. The NR1H and NR1I receptors: constitutive androstane receptor, pregnene X receptor, farnesoid X receptor alpha, farnesoid X receptor beta, liver X receptor alpha, liver X receptor beta, and vitamin D receptor. Pharmacol Rev. (2006) 58:742–59. doi: 10.1124/pr.58.4.6. PMID: 17132852

[10] Ma X Shah Y Cheung C Guo GL Feigenbaum L Krausz KW . The PREgnane X receptor gene-humanized mouse: a model for investigating drug-drug interactions mediated by cytochromes P450 3A. Drug Metab Dispos. (2007) 35:194–200. doi: 10.1124/dmd.106.012831. PMID: 17093002

[11] Xiao L Nickbarg E Wang W Thomas A Ziebell M Prosise WW . Evaluation of *in vitro* PXR-based assays and in silico modeling approaches for understanding the binding of a structurally diverse set of drugs to PXR. Biochem Pharmacol. (2011) 81:669–79. doi: 10.1016/j.bcp.2010.12.003. PMID: 21145880

[12] Daujat-Chavanieu M Gerbal-Chaloin S . Regulation of CAR and PXR expression in health and disease. Cells. (2020) 9(11):2395. doi: 10.3390/cells9112395. PMID: 33142929 PMC7692647

[13] Dutta M Lim JJ Cui JY . Pregnane X receptor and the gut-liver axis: a recent update. Drug Metab Dispos. (2022) 50:478–91. doi: 10.1124/dmd.121.000415. PMID: 34862253 PMC11022899

[14] Lehmann JM McKee DD Watson MA Willson TM Moore JT Kliewer SA . The human orphan nuclear receptor PXR is activated by compounds that regulate CYP3A4 gene expression and cause drug interactions. J Clin Invest. (1998) 102:1016–23. doi: 10.1172/jci3703. PMID: 9727070 PMC508967

[15] Synold TW Dussault I Forman BM . The orphan nuclear receptor SXR coordinately regulates drug metabolism and efflux. Nat Med. (2001) 7:584–90. doi: 10.1038/87912. PMID: 11329060

[16] Mottis A Mouchiroud L Auwerx J . Emerging roles of the corepressors NCoR1 and SMRT in homeostasis. Genes Dev. (2013) 27:819–35. doi: 10.1101/gad.214023.113. PMID: 23630073 PMC3650221

[17] Kodama S Koike C Negishi M Yamamoto Y . Nuclear receptors CAR and PXR cross talk with FOXO1 to regulate genes that encode drug-metabolizing and gluconeogenic enzymes. Mol Cell Biol. (2004) 24:7931–40. doi: 10.1128/mcb.24.18.7931-7940.2004. PMID: 15340055 PMC515037

[18] Kodama S Moore R Yamamoto Y Negishi M . Human nuclear pregnane X receptor cross-talk with CREB to repress cAMP activation of the glucose-6-phosphatase gene. Biochem J. (2007) 407:373–81. doi: 10.1042/bj20070481. PMID: 17635106 PMC2275060

[19] Pascussi JM Gerbal-Chaloin S Duret C Daujat-Chavanieu M Vilarem MJ Maurel P . The tangle of nuclear receptors that controls xenobiotic metabolism and transport: crosstalk and consequences. Annu Rev Pharmacol Toxicol. (2008) 48:1–32. doi: 10.1146/annurev.pharmtox.47.120505.105349. PMID: 17608617

[20] Ding X Staudinger JL . Repression of PXR-mediated induction of hepatic CYP3A gene expression by protein kinase C. Biochem Pharmacol. (2005) 69:867–73. doi: 10.1016/j.bcp.2004.11.025. PMID: 15710363

[21] Lichti-Kaiser K Xu C Staudinger JL . Cyclic AMP-dependent protein kinase signaling modulates pregnane x receptor activity in a species-specific manner. J Biol Chem. (2009) 284:6639–49. doi: 10.1074/jbc.m807426200. PMID: 19141612 PMC2652295

[22] Lichti-Kaiser K Brobst D Xu C Staudinger JL . A systematic analysis of predicted phosphorylation sites within the human pregnane X receptor protein. J Pharmacol Exp Ther. (2009) 331:65–76. doi: 10.1124/jpet.109.157180. PMID: 19617467 PMC2766221

[23] Rogers RS Parker A Vainer PD Elliott E Sudbeck D Parimi K . The interface between cell signaling pathways and pregnane X receptor. Cells. (2021) 10(11):3262. doi: 10.3390/cells10113262. PMID: 34831484 PMC8617909

[24] Staudinger JL Xu C Biswas A Mani S . Post-translational modification of pregnane x receptor. Pharmacol Res. (2011) 64:4–10. doi: 10.1016/j.phrs.2011.02.011. PMID: 21397695 PMC3111031

[25] Wang C Xu W Zhang Y Huang D Huang K . Poly(ADP-ribosyl)ated PXR is a critical regulator of acetaminophen-induced hepatotoxicity. Cell Death Dis. (2018) 9:819. doi: 10.1038/s41419-018-0875-4. PMID: 30050067 PMC6062506

[26] Kliewer SA Goodwin B Willson TM . The nuclear pregnane X receptor: a key regulator of xenobiotic metabolism. Endocr Rev. (2002) 23:687–702. doi: 10.1210/er.2001-0038. PMID: 12372848

[27] Tolson AH Wang H . Regulation of drug-metabolizing enzymes by xenobiotic receptors: PXR and CAR. Adv Drug Delivery Rev. (2010) 62:1238–49. doi: 10.1016/j.addr.2010.08.006. PMID: 20727377 PMC2991607

[28] Huang K Mukherjee S DesMarais V Albanese JM Rafti E Draghi AII . Targeting the PXR-TLR4 signaling pathway to reduce intestinal inflammation in an experimental model of necrotizing enterocolitis. Pediatr Res. (2018) 83:1031–40. doi: 10.1038/pr.2018.14. PMID: 29360809 PMC5959752

[29] Lan H Zhang Y Fan M Wu B Wang C . Pregnane X receptor as a therapeutic target for cholestatic liver injury. Drug Metab Rev. (2023) 55(4):371–87. doi: 10.1080/03602532.2023.2248680. PMID: 37593784

[30] Spruiell K Richardson RM Cullen JM Awumey EM Gonzalez FJ Gyamfi MA . Role of pregnane X receptor in obesity and glucose homeostasis in male mice. J Biol Chem. (2014) 289:3244–61. doi: 10.1074/jbc.m113.494575. PMID: 24362030 PMC3916528

[31] Sun L Sun Z Wang Q Zhang Y Jia Z . Role of nuclear receptor PXR in immune cells and inflammatory diseases. Front Immunol. (2022) 13:969399. doi: 10.3389/fimmu.2022.969399. PMID: 36119030 PMC9481241

[32] Zhou J Zhai Y Mu Y Gong H Uppal H Toma D . A novel pregnane X receptor-mediated and sterol regulatory element-binding protein-independent lipogenic pathway. J Biol Chem. (2006) 281:15013–20. doi: 10.1074/jbc.m511116200. PMID: 16556603 PMC4109972

[33] Xie W Tian Y . Xenobiotic receptor meets NF-kappaB, a collision in the small bowel. Cell Metab. (2006) 4:177–8. doi: 10.1016/j.cmet.2006.08.004. PMID: 16950133

[34] Gerbal-Chaloin S Iankova I Maurel P Daujat-Chavanieu M . Nuclear receptors in the cross-talk of drug metabolism and inflammation. Drug Metab Rev. (2013) 45:122–44. doi: 10.3109/03602532.2012.756011. PMID: 23330545

[35] Klepsch V Moschen AR Tilg H Baier G Hermann-Kleiter N . Nuclear receptors regulate intestinal inflammation in the context of IBD. Front Immunol. (2019) 10:1070. doi: 10.3389/fimmu.2019.01070. PMID: 31139192 PMC6527601

[36] Venkatesh M Mukherjee S Wang H Li H Sun K Benechet AP . Symbiotic bacterial metabolites regulate gastrointestinal barrier function via the xenobiotic sensor PXR and Toll-like receptor 4. Immunity. (2014) 41:296–310. doi: 10.1016/j.immuni.2014.06.014. PMID: 25065623 PMC4142105

[37] Dvorak Z Kopp F Costello CM Kemp JS Li H Vrzalová A . Targeting the pregnane X receptor using microbial metabolite mimicry. EMBO Mol Med. (2020) 12:e11621. doi: 10.15252/emmm.201911621 32153125 PMC7136958

[38] Flannigan KL Nieves KM Szczepanski HE Serra A Lee JW Alston LA . The pregnane X receptor and indole-3-propionic acid shape the intestinal mesenchyme to restrain inflammation and fibrosis. Cell Mol Gastroenterol Hepatol. (2023) 15:765–95. doi: 10.1016/j.jcmgh.2022.10.014. PMID: 36309199 PMC9883297

[39] Medzhitov R . Origin and physiological roles of inflammation. Nature. (2008) 454:428–35. doi: 10.1038/nature07201. PMID: 18650913

[40] Nathan C . Points of control in inflammation. Nature. (2002) 420:846–52. doi: 10.1038/nature01320. PMID: 12490957

[41] Netea MG Balkwill F Chonchol M Cominelli F Donath MY Giamarellos-Bourboulis EJ . A guiding map for inflammation. Nat Immunol. (2017) 18:826–31. doi: 10.1038/ni.3790. PMID: 28722720 PMC5939996

[42] Copeland S Warren HS Lowry SF Calvano SE Remick D Inflammation and the Host Response to Injury Investigators . Acute inflammatory response to endotoxin in mice and humans. Clin Diagn Lab Immunol. (2005) 12:60–7. doi: 10.1128/cdli.12.1.60-67.2005. PMID: 15642986 PMC540200

[43] Serhan CN Savill J . Resolution of inflammation: the beginning programs the end. Nat Immunol. (2005) 6:1191–7. doi: 10.1038/ni1276. PMID: 16369558

[44] Barton GM . A calculated response: control of inflammation by the innate immune system. J Clin Invest. (2008) 118:413–20. doi: 10.1172/jci34431. PMID: 18246191 PMC2214713

[45] Varela ML Mogildea M Moreno I Lopes A . Acute inflammation and metabolism. Inflammation. (2018) 41:1115–27. doi: 10.1007/s10753-018-0739-1. PMID: 29404872

[46] Chen L Deng H Cui H Fang J Zuo Z Deng J . Inflammatory responses and inflammation-associated diseases in organs. Oncotarget. (2018) 9:7204–18. doi: 10.18632/oncotarget.23208. PMID: 29467962 PMC5805548

[47] Takeda K Akira S . Toll-like receptors. Curr Protoc Immunol. (2007) Supplement 77(Chapter 14):14.12.1–14.12.13. doi: 10.1016/b0-12-443710-9/00729-8. PMID: 18432983

[48] Takeda K Akira S . Toll-like receptors. Curr Protoc Immunol. (2015) 109:14 12 1–14 12 10. doi: 10.1016/b0-12-443710-9/00729-8. PMID: 25845562

[49] Serhan CN . Resolution phase of inflammation: novel endogenous anti-inflammatory and proresolving lipid mediators and pathways. Annu Rev Immunol. (2007) 25:101–37. doi: 10.1146/annurev.immunol.25.022106.141647. PMID: 17090225

[50] Strober W Fuss I Mannon P . The fundamental basis of inflammatory bowel disease. J Clin Invest. (2007) 117:514–21. doi: 10.1172/jci30587. PMID: 17332878 PMC1804356

[51] Fukata M Arditi M . The role of pattern recognition receptors in intestinal inflammation. Mucosal Immunol. (2013) 6:451–63. doi: 10.1038/mi.2013.13. PMID: 23515136 PMC3730813

[52] McGuckin MA Eri R Simms LA Florin TH Radford-Smith G . Intestinal barrier dysfunction in inflammatory bowel diseases. Inflammation Bowel Dis. (2009) 15:100–13. doi: 10.1002/ibd.20539. PMID: 18623167

[53] Soderholm AT Pedicord VA . Intestinal epithelial cells: at the interface of the microbiota and mucosal immunity. Immunology. (2019) 158:267–80. doi: 10.1111/imm.13117. PMID: 31509239 PMC6856932

[54] Aggeletopoulou I Kalafateli M Tsounis EP Triantos C . Exploring the role of IL-1beta in inflammatory bowel disease pathogenesis. Front Med (Lausanne). (2024) 11:1307394. doi: 10.3389/fmed.2024.1307394. PMID: 38323035 PMC10845338

[55] Malik TF Aurelio DM . Extraintestinal manifestations of inflammatory bowel disease. In: Statpearls. Treasure Island (FL): StatPearls Publishing (2024). 33760556

[56] Rogler G Singh A Kavanaugh A Rubin DT . Extraintestinal manifestations of inflammatory bowel disease: Current concepts, treatment, and implications for disease management. Gastroenterology. (2021) 161:1118–32. doi: 10.1053/j.gastro.2021.07.042. PMID: 34358489 PMC8564770

[57] Vavricka SR Schoepfer A Scharl M Lakatos PL Navarini A Rogler G . Extraintestinal manifestations of inflammatory bowel disease. Inflammation Bowel Dis. (2015) 21:1982–92. doi: 10.1097/mib.0000000000000392. PMID: 26154136 PMC4511685

[58] Alatshan A Benko S . Nuclear receptors as multiple regulators of NLRP3 inflammasome function. Front Immunol. (2021) 12:630569. doi: 10.3389/fimmu.2021.630569. PMID: 33717162 PMC7952630

[59] Fiorucci S Carino A Baldoni M Santucci L Costanzi E Graziosi L . Bile acid signaling in inflammatory bowel diseases. Dig Dis Sci. (2021) 66:674–93. doi: 10.1007/s10620-020-06715-3. PMID: 33289902 PMC7935738

[60] Langmann T Moehle C Mauerer R Scharl M Liebisch G Zahn A . Loss of detoxification in inflammatory bowel disease: dysregulation of pregnane X receptor target genes. Gastroenterology. (2004) 127:26–40. doi: 10.1053/j.gastro.2004.04.019. PMID: 15236169

[61] Van den Bossche L Borsboom D Devriese S Van Welden S Holvoet T Dehairs J . Tauroursodeoxycholic acid protects bile acid homeostasis under inflammatory conditions and dampens Crohn’s disease-like ileitis. Lab Invest. (2017) 97:519–29. doi: 10.1038/labinvest.2017.6. PMID: 28165466

[62] Deuring JJ Li M Cao W Chen S Wang W de Haar C . Pregnane X receptor activation constrains mucosal NF-kappaB activity in active inflammatory bowel disease. PloS One. (2019) 14:e0221924. doi: 10.1371/journal.pone.0221924. PMID: 31581194 PMC6776398

[63] Kusunoki Y Ikarashi N Hayakawa Y Ishii M Kon R Ochiai W . Hepatic early inflammation induces downregulation of hepatic cytochrome P450 expression and metabolic activity in the dextran sulfate sodium-induced murine colitis. Eur J Pharm Sci. (2014) 54:17–27. doi: 10.1016/j.ejps.2013.12.019. PMID: 24413062

[64] Kusunoki Y Ikarashi N Matsuda S Matsukawa Y Kitaoka S Kon R . Expression of hepatic cytochrome P450 in a mouse model of ulcerative colitis changes with pathological conditions. J Gastroenterol Hepatol. (2015) 30:1618–26. doi: 10.1111/jgh.12966. PMID: 25867644

[65] Chen YH Wang JP Wang H Sun MF Wei LZ Wei W . Lipopolysaccharide treatment downregulates the expression of the pregnane X receptor, cyp3a11 and mdr1a genes in mouse placenta. Toxicology. (2005) 211:242–52. doi: 10.1016/j.tox.2005.03.011. PMID: 15869837

[66] Xie W Barwick JL Downes M Blumberg B Simon CM Nelson MC . Humanized xenobiotic response in mice expressing nuclear receptor SXR. Nature. (2000) 406:435–9. doi: 10.1038/35019116. PMID: 10935643

[67] Staudinger JL Goodwin B Jones SA Hawkins-Brown D MacKenzie KI LaTour A . The nuclear receptor PXR is a lithocholic acid sensor that protects against liver toxicity. Proc Natl Acad Sci USA. (2001) 98:3369–74. doi: 10.1073/pnas.051551698. PMID: 11248085 PMC30660

[68] Luan Z Wei Y Huo X Sun X Zhang C Ming W . Pregnane X receptor (PXR) protects against cisplatin-induced acute kidney injury in mice. Biochim Biophys Acta Mol Basis Dis. (2021) 1867:165996. doi: 10.1016/j.bbadis.2020.165996. PMID: 33127475

[69] Ye N Wang H Hong J Zhang T Lin C Meng C . PXR mediated protection against liver inflammation by Ginkgolide A in tetrachloromethane treated mice. Biomol Ther (Seoul). (2016) 24:40–8. doi: 10.4062/biomolther.2015.077. PMID: 26759700 PMC4703351

[70] Zhang X Wang Y Ma Z Liang Q Tang X Hu D . Tanshinone IIA ameliorates dextran sulfate sodium-induced inflammatory bowel disease via the pregnane X receptor. Drug Des Devel Ther. (2015) 9:6343–62. doi: 10.2147/dddt.s79388. PMID: 26674743 PMC4676510

[71] Zhang X Ma Z Liang Q Tang X Hu D Liu C . Tanshinone IIA exerts protective effects in a LCA-induced cholestatic liver model associated with participation of pregnane X receptor. J Ethnopharmacol. (2015) 164:357–67. doi: 10.1016/j.jep.2015.01.047. PMID: 25660334

[72] Gwag T Meng Z Sui Y Helsley RN Park SH Wang S . Non-nucleoside reverse transcriptase inhibitor efavirenz activates PXR to induce hypercholesterolemia and hepatic steatosis. J Hepatol. (2019) 70:930–40. doi: 10.1016/j.jhep.2018.12.038. PMID: 30677459 PMC6462244

[73] Meng Z Gwag T Sui Y Park SH Zhou X Zhou C . The atypical antipsychotic quetiapine induces hyperlipidemia by activating intestinal PXR signaling. JCI Insight. (2019) 4(3):e125657. doi: 10.1172/jci.insight.125657. PMID: 30728326 PMC6413802

[74] Sui Y Meng Z Chen J Liu J Hernandez R Gonzales MB . Effects of dicyclohexyl phthalate exposure on PXR activation and lipid homeostasis in mice. Environ Health Perspect. (2021) 129:127001. doi: 10.1289/ehp9262. PMID: 34851150 PMC8634903

[75] El Marjou F Janssen KP Chang BHJ Li M Hindie V Chan L . Tissue-specific and inducible Cre-mediated recombination in the gut epithelium. Genesis. (2004) 39:186–93. doi: 10.1002/gene.20042. PMID: 15282745

[76] Amini SE Bresson SE Ruzzin J . Mice lacking intestinal Nr1i2 have normal intestinal homeostasis under steady-state conditions and are not hypersensitive to inflammation under lipopolysaccharide treatment. FASEB J. (2023) 37:e23117. doi: 10.1096/fj.202301126. PMID: 37490003

[77] Deguchi Y Andoh A Yagi Y Bamba S Inatomi O Tsujikawa T . The S1P receptor modulator FTY720 prevents the development of experimental colitis in mice. Oncol Rep. (2006) 16:699–703. doi: 10.3892/or.16.4.699 16969482

[78] Nazari-Khanamiri F Jafari A Esmaeilzadeh Z Ghasemnejad-Berenji M . Biochemical and histopathological evidence for beneficial effects of Empagliflozin pretreatment on acetic acid-induced colitis in rats. BMC Gastroenterol. (2023) 23:332. doi: 10.1186/s12876-023-02958-2. PMID: 37759154 PMC10523708

[79] Zhou C Tabb MM Nelson EL Grün F Verma S Sadatrafiei A . Mutual repression between steroid and xenobiotic receptor and NF-kappaB signaling pathways links xenobiotic metabolism and inflammation. J Clin Invest. (2006) 116:2280–9. doi: 10.1172/jci26283. PMID: 16841097 PMC1501109

[80] Abualsunun WA Piquette-Miller M . Involvement of nuclear factor kappaB, not pregnane X receptor, in inflammation-mediated regulation of hepatic transporters. Drug Metab Dispos. (2017) 45:1077–83. doi: 10.1124/dmd.117.076927. PMID: 28778997

[81] Shah YM Ma X Morimura K Kim I Gonzalez FJ . Pregnane X receptor activation ameliorates DSS-induced inflammatory bowel disease via inhibition of NF-kappaB target gene expression. Am J Physiol Gastrointest Liver Physiol. (2007) 292:G1114–22. doi: 10.1201/9780429322570-5 17170021

[82] Kozik AJ Nakatsu CH Chun H Jones-Hall YL . Age, sex, and TNF associated differences in the gut microbiota of mice and their impact on acute TNBS colitis. Exp Mol Pathol. (2017) 103:311–9. doi: 10.1016/j.yexmp.2017.11.014. PMID: 29175304

[83] Kim YS Unno T Kim BY Park MS . Sex differences in gut microbiota. World J Mens Health. (2020) 38:48–60. doi: 10.5534/wjmh.190009. PMID: 30929328 PMC6920072

[84] Little M Dutta M Li H Matson A Shi X Mascarinas G . Understanding the physiological functions of the host xenobiotic-sensing nuclear receptors PXR and CAR on the gut microbiome using genetically modified mice. Acta Pharm Sin B. (2022) 12:801–20. doi: 10.21203/rs.3.rs-36593/v1. PMID: 35256948 PMC8897037

[85] Dubrac S Elentner A Ebner M Horejs-Hoeck J Schmuth M . Modulation of T lymphocyte function by the pregnane X receptor. J Immunol. (2010) 184:2949–57. doi: 10.4049/jimmunol.0902151. PMID: 20173028

[86] Sun M Cui W Woody SK Staudinger JL . Pregnane X receptor modulates the inflammatory response in primary cultures of hepatocytes. Drug Metab Dispos. (2015) 43:335–43. doi: 10.1124/dmd.114.062307. PMID: 25527709 PMC4352581

[87] Xuan LN Hu ZX Jiang ZF Zhang C Sun XW Ming WH . Pregnane X receptor (PXR) deficiency protects against spinal cord injury by activating NRF2/HO-1 pathway. CNS Neurosci Ther. (2023) 29:3460–78. doi: 10.1111/cns.14279. PMID: 37269088 PMC10580351

[88] Erickson SL Alston L Nieves K Chang TKH Mani S Flannigan KL . The xenobiotic sensing pregnane X receptor regulates tissue damage and inflammation triggered by C difficile toxins. FASEB J. (2020) 34:2198–212. doi: 10.1096/fj.201902083rr. PMID: 31907988 PMC7027580

[89] Qiu Z Cervantes JL Cicek BB Mukherjee S Venkatesh M Maher LA . Pregnane X receptor regulates pathogen-induced inflammation and host defense against an intracellular bacterial infection through Toll-like receptor 4. Sci Rep. (2016) 6:31936. doi: 10.1038/srep31936. PMID: 27550658 PMC4994038

[90] Dou W Mukherjee S Li H Venkatesh M Wang H Kortagere S . Alleviation of gut inflammation by Cdx2/Pxr pathway in a mouse model of chemical colitis. PloS One. (2012) 7:e36075. doi: 10.1371/journal.pone.0036075. PMID: 22815676 PMC3398007

[91] Cheng J Shah YM Ma X Pang X Tanaka T Kodama T . Therapeutic role of rifaximin in inflammatory bowel disease: clinical implication of human pregnane X receptor activation. J Pharmacol Exp Ther. (2010) 335:32–41. doi: 10.1124/jpet.110.170225. PMID: 20627999 PMC2957776

[92] Cheng J Fang ZZ Nagaoka K Okamoto M Qu A Tanaka N . Activation of intestinal human pregnane X receptor protects against azoxymethane/dextran sulfate sodium-induced colon cancer. J Pharmacol Exp Ther. (2014) 351:559–67. doi: 10.1124/jpet.114.215913. PMID: 25277138 PMC4244584

[93] Egusquiza RJ Ambrosio ME Wang SG Kay KM Zhang C Lehmler HJ . Evaluating the role of the steroid and xenobiotic receptor (SXR/PXR) in PCB-153 metabolism and protection against associated adverse effects during perinatal and chronic exposure in mice. Environ Health Perspect. (2020) 128:47011. doi: 10.1289/ehp6262. PMID: 32352317 PMC7228131

[94] Guo GL Moffit JS Nicol CJ Ward JM Aleksunes LA Slitt AL . Enhanced acetaminophen toxicity by activation of the pregnane X receptor. Toxicol Sci. (2004) 82:374–80. doi: 10.1093/toxsci/kfh286. PMID: 15456926

[95] Xie W Radominska-Pandya A Shi Y Simon CM Nelson MC Ong ES . An essential role for nuclear receptors SXR/PXR in detoxification of cholestatic bile acids. Proc Natl Acad Sci USA. (2001) 98:3375–80. doi: 10.1073/pnas.051014398. PMID: 11248086 PMC30661

[96] Xie Y Xu M Deng M Li Z Wang P Ren S . Activation of pregnane X receptor sensitizes mice to hemorrhagic shock-induced liver injury. Hepatology. (2019) 70:995–1010. doi: 10.1002/hep.30691. PMID: 31038762 PMC6717545

[97] He J Gao J Xu M Ren S Stefanovic-Racic M O'Doherty RM . PXR ablation alleviates diet-induced and genetic obesity and insulin resistance in mice. Diabetes. (2013) 62:1876–87. doi: 10.2337/db12-1039. PMID: 23349477 PMC3661619

[98] Karpale M Kummu O Kärkkäinen O Lehtonen M Näpänkangas J Herfurth UM . Pregnane X receptor activation remodels glucose metabolism to promote NAFLD development in obese mice. Mol Metab. (2023) 76:101779. doi: 10.1016/j.molmet.2023.101779. PMID: 37467962 PMC10415798

[99] Kim S Choi S Dutta M Asubonteng JO Polunas M Goedken M . Pregnane X receptor exacerbates nonalcoholic fatty liver disease accompanied by obesity- and inflammation-prone gut microbiome signature. Biochem Pharmacol. (2021) 193:114698. doi: 10.1016/j.bcp.2021.114698. PMID: 34303710 PMC9135326

[100] Garg A Zhao A Erickson SL Mukherjee S Lau AJ Alston L . Pregnane X receptor activation attenuates inflammation-associated intestinal epithelial barrier dysfunction by inhibiting cytokine-induced myosin light-chain kinase expression and c-Jun N-terminal kinase 1/2 activation. J Pharmacol Exp Ther. (2016) 359:91–101. doi: 10.1124/jpet.116.234096. PMID: 27440420 PMC5034705

[101] Hudson G Flannigan KL Pulakazhi Venu VK Alston L Sandall CF MacDonald JA . Pregnane X receptor activation triggers rapid ATP release in primed macrophages that mediates NLRP3 inflammasome activation. J Pharmacol Exp Ther. (2019) 370:44–53. doi: 10.1124/jpet.118.255679 PMC6542184 31004077

[102] Uehara D Tojima H Kakizaki S Yamazaki Y Horiguchi N Takizawa D . Constitutive androstane receptor and pregnane X receptor cooperatively ameliorate DSS-induced colitis. Dig Liver Dis. (2019) 51:226–35. doi: 10.1016/j.dld.2018.10.008. PMID: 30442521

[103] Yan T Luo Y Xia Y Hamada K Wang Q Yan N . St. John's Wort alleviates dextran sodium sulfate-induced colitis through pregnane X receptor-dependent NFκB antagonism. FASEB J. (2021) 35:e21879. doi: 10.1096/fj.202001098R 34644426 PMC10167919

