# Supplementary material for: Pregnane X receptor (NR1I2) deficiency in mice reveals context-dependent regulation of inflammatory homeostasis
Source: Front Immunol. 2026 May 12;17:1761552. doi: 10.3389/fimmu.2026.1761552 (PMC13201179; doi:10.3389/fimmu.2026.1761552)
Supplement: Supplementary file 1 [file DataSheet1.docx]

**Supplementary Table 1. Comparative *in vivo* phenotypic map of inflammatory susceptibility in *Nr1i2* deficient mouse models.** This table summarizes the inflammatory and tissue injury phenotypes reported in whole-body knockout (Xie, Staudinger and Luan models), whole-body knockdown (siRNA and shRNA), and tissue-specific *Nr1i2*-deficient mice, including intestinal epithelial-specific (*Nr1i2^ΔIEC^*), intestinal fibroblast-specific (*Nr1i2^ΔCol1a2^*), and hepatocyte-specific (*Nr1i2^ΔHep^*) models. Phenotypes are presented across basal conditions and major experimental inflammatory contexts, including bacterial toxin induced injury (e.g., LPS, TcdA/B), chemically induced colitis (DSS acute phase and post injury healing/fibrosis), other xenobiotic or chemical injuries, experimental gut injury models (e.g., necrotizing enterocolitis (NEC), isc hemia-reperfusion (I/R)), and high-fat diet (HFD)-induced liver inflammation. Only in vivo phenotypes are included in this figure; *ex vivo* and *in vitro* findings are not represented to ensure direct comparability of systemic inflammatory responses. Arrows indicate direction of phenotypic change relative to control mice: ↗ increased susceptibility or severity; ↘ decreased susceptibility or severity; ≈ no significant change; NA not assessed *in vivo* in the cited studies; (*) recombination was induced by intraperitoneal 4-OHT injections; ∆ deletion.

| **Model and Genetic Strategy** | **Basal intestinal homeostasis** | **Bacterial toxins (TcdA/B, LPS)** | **Chemical colitis (DSS) acute phase** | **Post-colitis repair/fibrosis (DSS healing)** | **Other chemical injury (indomethacin, anti-CD3, PCB)** | **Experimental injury**  **(NEC, I/R)** | **Diet/metabolic inflammation (HFD liver)** |
| --- | --- | --- | --- | --- | --- | --- | --- |
| ***Xie et al.* Whole-body KO Model (*Nr1i2^−/−^*)**  ***∆ Exon 2-3 DBD disruption*** | **↗** | **↗** | **≈** | **↗** | **↗** | **↗** | **↘** |
| ***Staudinger et al.* Whole-body KO Model (*Nr1i2^−/−^*)**  ***∆ Exon 1 full gene disruption*** | **↗** | **↗** | **≈** | **↗** | **↗** | **↗** | **↘** |
| ***Luan et al.* Whole-body KO Model (*Nr1i2^−/−^*)**  ***∆ Exon 3***  ***Kidney validated*** | **NA** | **NA** | **NA** | **NA** | **NA** | **NA** | **NA** |
| **siRNA Whole-body KD Model (*Nr1i2-^KD^*)**  ***Transient systemic knockdown*** | **NA** | **NA** | **↗** | **NA** | **NA** | **NA** | **NA** |
| **shRNA Whole-body KD Model (*Nr1i2-^KD^*)**  **Stable systemic knockdown** | **NA** | **NA** | **NA** | **NA** | **NA** | **NA** | **NA** |
| **Intestinal Epithelial-specific KO (*Nr1i2^ΔIEC^*)**  ***Villin-Cre*** | **≈** | **≈** | **≈** | **≈** | **NA** | **NA** | **NA** |
| **Intestinal Fibroblast-specific KO (Nr1i2^ΔCol1a2^)**  ***CoI1a2-CreERT2**** | **NA** | **NA** | **≈** | **↗** | **NA** | **NA** | **NA** |
| **Hepatocyte-specific KO (*Nr1i2^ΔHep^*)**  ***Albumin-Cre*** | **NA** | **NA** | **NA** | **NA** | **NA** | **NA** | **↘/≈** |
